# Prevention of colonic neoplasia with polyethylene glycol: A short term randomized placebo-controlled double-blinded trial

**DOI:** 10.1371/journal.pone.0193544

**Published:** 2018-04-04

**Authors:** Ramesh K. Wali, Laura Bianchi, Sonia Kupfer, Mart De La Cruz, Borko Jovanovic, Christopher Weber, Michael J. Goldberg, L. M. Rodriguez, Raymond Bergan, David Rubin, Mary Beth Tull, Ellen Richmond, Beth Parker, Seema Khan, Hemant K. Roy

**Affiliations:** 1 Department of Medicine, Boston University Medical Center, Boston, MA, United States of America; 2 Department of Medicine, NorthShore University HealthSystem, Evanston, IL, United States of America; 3 Department of Medicine, University of Chicago, Chicago, IL, United States of America; 4 Department of Medicine, Northwestern University, Chicago, IL, United States of America; 5 National Cancer Institute, Rockville, MD, United States of America; 6 Department of Medicine, Oregon Health & Science University, Portland, OR, United States of America; University Hospital Llandough, UNITED KINGDOM

## Abstract

Chemoprevention represents an attractive modality against colorectal cancer (CRC) although widespread clinical implementation of promising agents (e.g. aspirin/NSAIDS) have been stymied by both suboptimal efficacy and concerns over toxicity. This highlights the need for better agents. Several groups, including our own, have reported that the over-the-counter laxative polyethylene glycol (PEG) has remarkable efficacy in rodent models of colon carcinogenesis. In this study, we undertook the first randomized human trial to address the role of PEG in prevention of human colonic neoplasia. This was a double-blind, placebo-controlled, three-arm trial where eligible subjects were randomized to 8g PEG-3350 (n = 27) or 17g PEG-3350 (n = 24), or placebo (n = 24; maltodextrin) orally for a duration of six months. Our initial primary endpoint was rectal aberrant crypt foci (ACF) but this was changed during protocol period to rectal mucosal epidermal growth factor receptor (EGFR). Of the 87 patients randomized, 48 completed study primary endpoints and rectal EGFR unchanged PEG treatment. Rectal ACF had a trend suggesting potentially reduction with PEG treatment (pre-post change 1.7 in placebo versus -0.3 in PEG 8+ 17g doses, p = 0.108). Other endpoints (proliferation, apoptosis, expression of SNAIL and E-cadherin), previously noted to be modulated in rodent models, appeared unchanged with PEG treatment in this clinical trial. We conclude that PEG was generally well tolerated with the trial failing to meet primary efficacy endpoints. However, rectal ACFs demonstrated a trend (albeit statistically insignificant) for suppression with PEG. Moreover, all molecular assays including EGFR were unaltered with PEG underscoring issues with lack of translatability of biomarkers from preclinical to clinical trials. This data may provide the impetus for future clinical trials on PEG using more robust biomarkers of chemoprevention.

**Trial registration:** ClinicalTrials.gov NCT00828984

## Introduction

In 2018, it is estimated that there will be 140,250 new colorectal cancer (CRC) cases in the U.S. resulting in 50,630 deaths [1]. Since symptoms are generally a harbinger of advanced incurable disease, efforts have focused on early detection through population screening which has been associated with a modest decrease in CRC mortality over the last decade. However, despite performing approximately 15 million colonoscopies a year along with a number of patients utilizing fecal tests (fecal immunohistochemistry, fecal DNA etc.) [2], CRC still ranks as the second leading cause of cancer deaths underscoring the need for more effective strategies [1].

Chemoprevention has emerged as a highly promising strategy since Sporn coined the term three decades ago [3]. There are a myriad of agents that have been shown to be active against CRC in cell culture, animal models, epidemiological studies and randomized controlled trials [4]. Aspirin is probably the best validated prevention agent with studies consistently showing a ~30–50% CRC risk reduction [5]. Indeed, aspirin has recently been sanctioned by US Preventive Services Task Force (USPSTF) for cardiovascular and CRC prevention, but this grade B recommendation is only for adults aged 50 to 59 years who have a 10% or greater 10-year CVD risk. Most of the benefit appears related to cardiovascular risk reduction. When considering only CRC prevention, the USPSTF previously reported that the risks of aspirin outweighed its benefit [6, 7]. Efforts to identify aspirin/NSAID responders to CRC have been complicated by a myriad of putative targets including COX 2, 15-PDGH etc., making identifying the optimal targets challenging. Thus, for chemoprevention to be clinically viable for most of the population, safer and more efficacious agents are required.

Two decades ago, Corpet, Paraud and colleagues provided intriguing reports that polyethylene glycol (PEG) was remarkably effective in CRC prevention [8, 9]. Polyethylene glycol exists as a polymer of a variety of molecular weights with agents tested in chemoprevention ranging from PEG 800 to 15,000. Indeed, a report suggested that PEG 8000 (molecular weight) was ranked as the most effective in comparison to other known agent in chemoprevention of experimental colon carcinogenesis [10]. Oral PEG is an osmotic laxative found in a number of over-the-counter (OTC) medications against constipation. Oral PEG is an attractive agent given its lack of absorption thus limiting systemic toxicity. Several groups, including our own, have replicated this work in rat the carcinogen-models [11, 12]. Furthermore, we recapitulated these findings in genetic rodent models (MIN mouse [13] and Pirc rat [14, 15]), which has germline truncations in the adenomatous polyposis coli (APC) tumor suppressor gene, the most common initiating driver mutation in human colorectal carcinogenesis. From a mechanistic perspective, our laboratory has demonstrated that PEG 8000 downregulated epidermal growth factor receptor (EGFR) with concomitant downregulation of SNAIL, induction of E-cadherin leading to β-catenin sequestration away from the nucleus and hence suppressing TCF/LEF-1 transcriptional activity [14]. Furthermore, we and others have shown induction of apoptosis and cell cycle arrest (potentially related to p21 cip/waf induction) [16].

While the preclinical data provides compelling evidence of the of CRC preventive effects of PEG (~20 publications by at least 4 independent groups [8, 10, 14, 15]) the only clinical data available is from a case-control study that had a number of methodological shortcomings [17]. Since epidemiological studies are often confounded by the indications for use of PEG (i.e. the underlying constipation) and lack of reliable data on over-the-counter (OTC) laxative consumption, robust clinical insights would mandate an interventional trial. Given that the typical adenoma prevention trial would be long (>3–5 years) and expensive, we designed a shorter term study with intermediate biomarker endpoints. Typically, the detection of aberrant crypt foci (ACF) using chromoendoscopy has been utilized to test anti-neoplastic effects of sulindac, celecoxib, and other agents [18–20]. Therefore, in our phase IIB study placebo-controlled randomized trial using two doses of PEG 3350, we initially chose the primary endpoints of ACF along with EGFR expression (although during the study we changed the primary endpoint to EGFR given recruitment considerations). In conjunction we employed other more exploratory endpoints (proliferation, apoptosis, expression of SNAIL and e-cadherin).

## Materials & methods

### Study design

This was a double-blind, placebo-controlled, three-arm trial (www.clinicaltrial.gov NCT00828984) where eligible subjects were randomized to 8g PEG-3350 or 17g PEG-3350 (Miralax OTC, which was repackaged into sachets), or placebo (maltodextrin sachets) for a duration of six months. The first subject registered for the study on 12/30/2009. The last subject completed follow-up on 10/7/2014. The study drug was administered orally, once daily. The study was conducted at Northshore University Healthcare and University of Chicago. International ethical and scientific standards of Good Clinical Practice (GCP) for trial design, conduct, monitoring, and reporting were rigorously followed. All subjects gave written informed consent following approval by the Institutional Review Board (IRB) of each academic institution. Initially, we had specified ACF as the primary endpoint but given a relatively lower number of rectal ACF than anticipated, we changed the primary endpoint to EGFR expression. The National Cancer Institute (NCI) Division of Cancer Prevention (DCP) performed scientific and statistical reviews of the protocol, sponsored and monitored the trial and housed the data through Westat (Rockville, MD). Polyethylene Glycol-3350 (8g and 17g) and matched placebo sachets were prepared by the NCI. The data were then locked, un-blinded and released to the study biostatistician (Dr. Jovanovic) who analyzed all the pertinent data.

### Study subjects

Inclusion criteria included patients undergoing surveillance colonoscopy for a previous history of adenoma or CRC within 6 years and greater than five ACF on entry colonoscopy. However, this proved to be a barrier to recruitment since most screened participants had fewer than 5 ACF and therefore were dropped as an entry criterion early in the recruitment phase. Exclusion criteria included age >70 years, baseline diarrhea, active malignancy, use of anticoagulants, NSAIDS, or chemotherapy. Also excluded were those with bone marrow, liver or renal dysfunction, coagulopathy or a history of invasive malignancy within the past year. In addition, patients with incomplete colonoscopy or with inadequate bowel preparation were also excluded. Baseline colonoscopy preparation involved a 4 L polyethylene glycol purge (e.g., Golytely or NuLytely); six biopsies of endoscopically normal rectum were performed (3 biopsies were formalin fixed for the biomarker expression analyses and the other 3 were snap frozen for RT-PCR or ELISA assays). Subjects were randomized 1:1:1 to placebo or 8g PEG-3350 or 17g PEG-3350 daily. Six-month biopsy was via fiexible sigmoidoscopy, using an 8oz. magnesium citrate purgative.

### Aberrant crypt foci (ACF) determination

ACF are the first identifiable morphological manifestation during CRC carcinogenesis and are reliable markers of field carcinogenesis. The translation from its role as a stalwart of rodent chemoprevention trials to human studies was facilitated by the advent of chromo-endoscopy [18]. Consenting subjects underwent completion of usual colonoscopy with Olympus 160 generation endoscope by board-certified gastroenterologists who had received standardized ACF training. Upon scope withdrawal, the rectum was sprayed with ~ 200 ml of 0.25% methylene blue solution, rinsed with large volumes of distilled water and the total number of rectal ACF were scored [18, 20, 21].

### Immunohistochemical (IHC) analyses of biomarkers

The biopsy sections were subjected to IHC analysis to determine the expression of several cellular biomarkers following PEG treatment. These included EGFR, Ki-67, SNAIL, Cleaved Caspase-3 and E-cadherin, all of which have previously been reported by our group to be modulated by PEG in cell culture and animal models [14]. For these studies, 2–3 rectal biopsies were formalin fixed, paraffin embedded, sectioned and subjected to the IHC evaluation by standard techniques using appropriate primary antibodies [anti-Ki67 (1:250; AbCam, Cambridge, MA), anti-EGFR (1:200; Santa Cruz, CA, anti-Cleaved Caspase 3 (1: 100; Cell Signaling Technology, Danvers, MA)], anti-SNAIL (1:100; Snai1–T18; Santa Cruz, CA) and anti-E-cadherin (1:250; Cell Signaling Technology) followed by appropriate biotinylated secondary antibodies. The antigen-antibody complexes were detected with the Vectastatin Elite ABC kit (Vector Laboratories). For negative controls, sections were processed in the absence of the primary antibodies. IHC sections were scored by the study pathologist (CW), who was blinded to the study group. A semi- quantitative scale was used to evaluate immunoreactivity of epithelial cells. The extent of staining was graded and scored as 0 (negative staining); 1+ (10% stained cells), 2+ (10–50% stained cells), and 3+ (50% stained cells).

### ELISA to measure EGFR protein

To supplement EGFR IHC, we performed an ELISA on 1–3 snap-frozen rectal biopsies which were pooled. Biopsies were incubated in a uticell extraction buffer (20-30ml; Invitrogen Cat # FNN0011) supplemented with 1 mM PMSF (Invitrogen) protease inhibitor cocktail (Sigma Chemicals) for 30–45 min at 4°C and then then centrifuged at 13,000 rpm for 10 min. The supernatant underwent protein estimation with Quant-iT assay kit (Invitrogen). For EGFR quantitation, 10 μg protein lysate was assessed with Invitrogen EGFR ELISA kit (KHR9061) according to the manufacturer’s directions.

### EGFR & SNAIL mRNA

Freshly isolated rectal biopsies (1–2) from control and PEG treated subjects were subjected to RT-PCR for mRNA expression of EGFR and SNAIL. Briefly, RNA was extracted with TRI Reagent (Sigma) as previously described [25]. The cDNA was synthesized using 2μg of RNA and Superscript RT (Invitrogen Life Technologies, CA). Normalization was performed to GAPDH expression.

### Missing data

**Fig 1:**
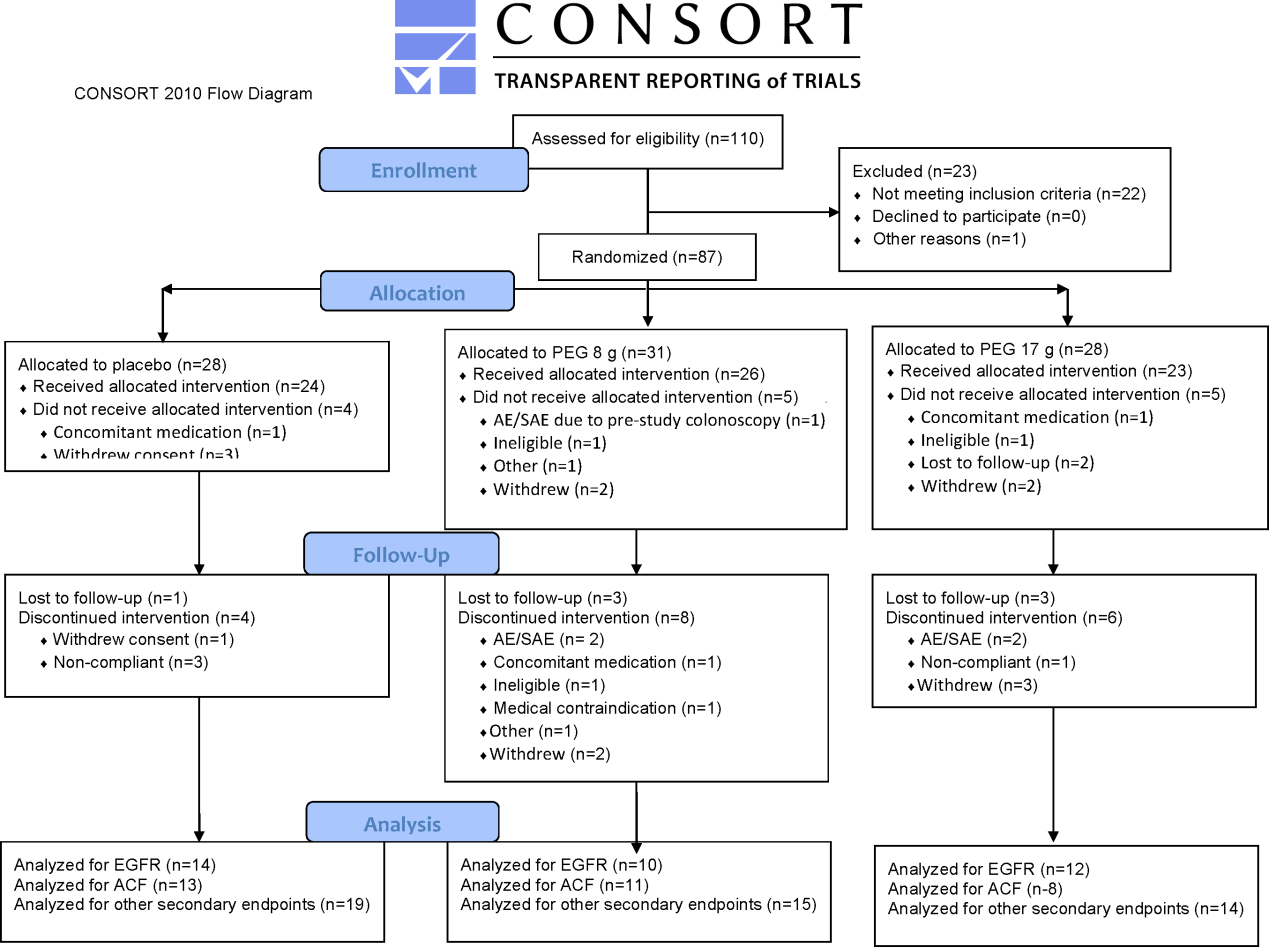
CONSORT diagram.

The number of participants completing intervention is shown in Fig 1 CONSORT Diagram but unfortunately biomarker data is not available on all participants. Missing data resulted from an institutional move of the investigators processing the samples from Chicago to Boston (RKW, MD and HKR). During transportation there was a failure of a -80°C freezer where samples were stored which resulted in lost samples. Also, change in personnel was responsible for some patients not undergoing rectal ACF analysis once it was relegated to a secondary endpoint.

### Statistical analysis

Means, SDs and ranges were computed for continuous demographic variables and for biomarkers at baseline and at 6 months. The within-subject difference between baseline and 6 months in the main outcomes of ACF and biomarkers was computed by treatment group (PEG-3350, placebo). Differences between groups (PEG-3350, placebo) were assessed via two-sample Wilcoxon–Mann–Whitney non-parametric tests for all biomarkers, and all reported p values are 2-sided. Frequencies and percentages were reported for categorical demographic variables and differences between treatment groups for these variables were evaluated via Fisher’s exact test. For regression plots, non-parametric Spearman correlation coefficient, and the corresponding non-parametric test, provided the corresponding two-sided p-value. Regression line slopes were computed using non-parametric correlation. All p-values are unadjusted and were based on all available data. Statistical analyses and graphics used SAS V.9.3 and R 2.15 software packages.

## Results

### Participant characteristics

The demographics of subjects who were randomized between placebo and two PEG doses, and initiated study treatment, were similar (Table 1). Patient recruitment was carried out as noted in the Fig 1 CONSORT Diagram. PEG was very well tolerated by the subjects with little attrition in patients given either PEG doses. Compliance was excellent (>80% doses taken) as gauged by medication (sachet) count and patient interviews.

**Table 1:** Demographics of study subjects.

	Placebo	PEG 8 g daily	PEG 17 g daily
	(*n* = 24)	(*n* = 27)	(*n* = 24)
Age, years (mean, range)	59.0 (36–74)	63.4 (46–80)	58.7 (30–72)
Male	13 (54.2%)	13 (48.1%)	12 (50.0%)
Race: Asian	2 (8.3%)	0 (0.0%)	0 (0.0%)
Race: Black or African American	0 (0.0%)	4 (14.8%)	4 (16.7%)
Race: White	22 (91.7%)	23 (85.2%)	20 (8.3%)
Ethnicity: Hispanic or Latino	0 (0.0%)	2 (7.4%)	1 (4.2%)
Smoking Status: Current	1 (4.2%)	0 (0.0%)	4 (16.7%)
Smoking Status: Past	11 (45.8%)	15 (55.6%)	11 (45.8%)
Smoking Status: Unknown	0 (0.0%)	0 (0.0%)	1 (4.2%)
BMI (mean, range)	28.1 (19.5–47.0)	28.5 (20.4–49.4)	29.5 (21.7–44.2)
Current use of NSAIDs: Yes	4 (16.7%)	6 (22.2%)	1 (4.2%)
Current use of NSAIDs: Unknown	0 (0.0%)	0 (0.0%)	1 (4.2%)

### PEG decreases rectal ACF numbers

**Fig 2:**
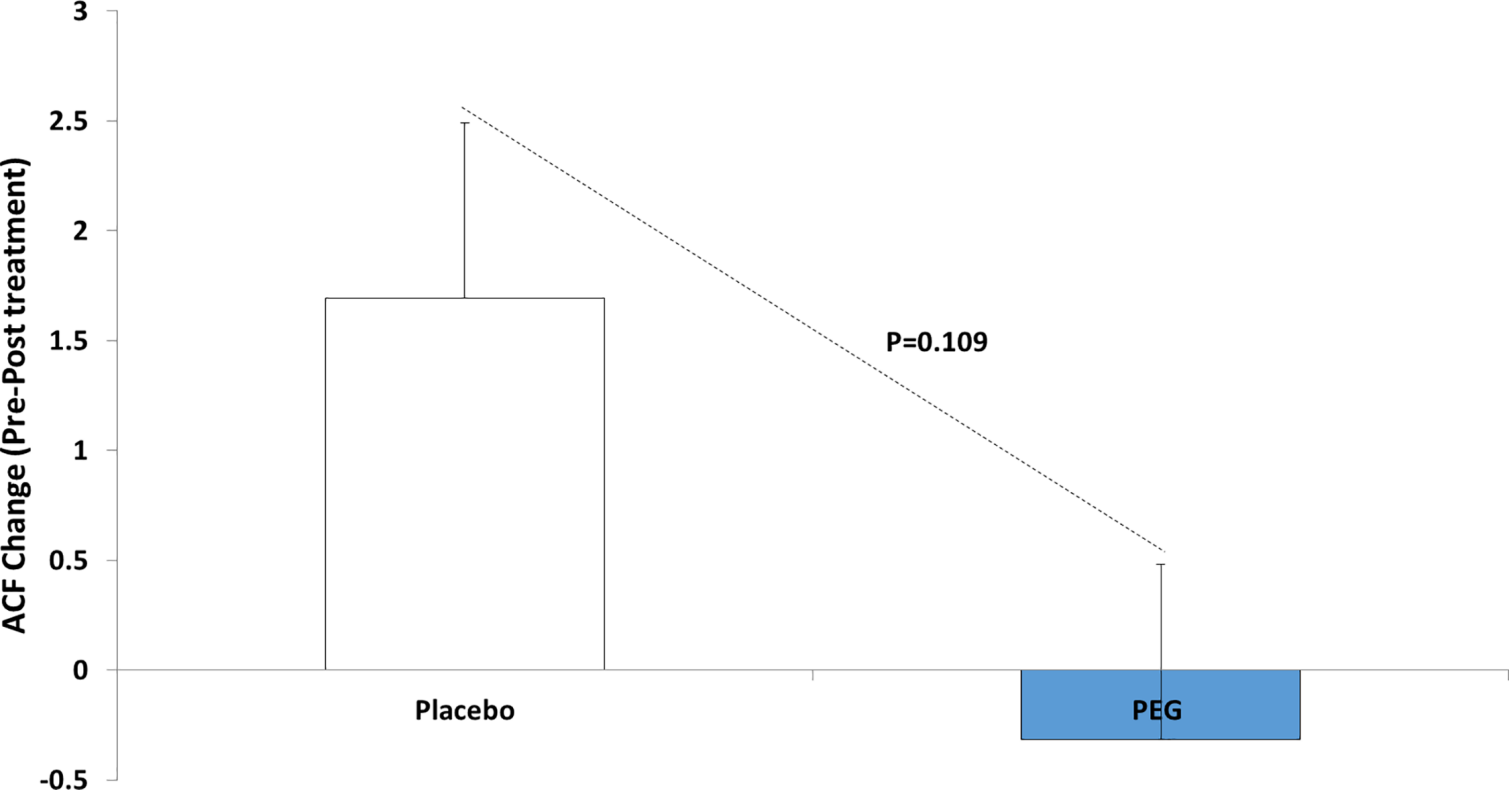
Reduction in the number of rectal ACF after PEG treatment.

Rectal ACF were used as an endpoint as they are well validated markers of neoplasia throughout the colon (i.e. marker of field carcinogenesis) [18]. As discussed previously, our initial primary endpoint for the study was ACF but given the difficulty of finding patients with ≥ 5 ACF (initially proposed in the inclusion criteria), we dropped this as an inclusion criterion and decided to use EGFR expression as the studies primary endpoint with rectal ACF number serving as secondary endpoint. Since the number of participants evaluable at each dose level was small (11 and 8 respectively), we pooled data from the two PEG dose groups 8 g and 17 g, which are shown in Table 2 and Fig 2. The data for each dose level separately are shown in S1 Table. As shown in Fig 2, there was a trend towards decrease in the number of rectal ACF in subjects treated with PEG 3350 (n = 19) compared to untreated (n = 13), with a p-value of 0.109. The effect of the 17 g dose was numerically larger than that of the 8 g dose (a decrease of 0.9 ACF at the higher dose and 0.1 ACF at the lower dose, see S1 Table.

**Table 2:** Changes in mean values of biomarkers in treated and placebo groups, with high and low PEG doses combined.

		Control (Placebo)					Intervention (8 g or 17 g PEG)					
		Pre-treatment	Post-treatment	n	change	p1^a^	Pre-treatment	Post-treatment	n	change	p1^a^	p2^b^
Rectal ACF (number/rectum)	Mean	2.5	4.2	13	1.7	0.148	5.4	5.1	19	0.3	0.598	0.109
	Range	[0, 13]	[0, 11]				[0, 9]	[0, 16]				
EGFR IHC (staining intensity)	Mean	1.93	2.14	14	0.2	0.256	2.0	1.9	22	0.1	0.621	0.280
	Range	[1, 3]	[1, 3]				[1, 3]	[0, 3]				
EGFR mRNA (fold-change)	Mean	1.76	5.04	13	2.7	0.023	1.8	3.9	21	2.1	0.054	0.620
	Range	[-6.27, 7.18]	[0.05, 7.17]				[-8.28, 7.56]	[-8.4, 8.4]				
EGFR ELISA (ng/100g protein)	Mean	3.09	3.75	6	0.7	0.173	3.9	4.0	10	0.1	0.721	0.448
	Range	[0.4, 6.4]	[2.2, 6.6]				[-0.48, 7.29]	[0.1, 8.3]				
SNAIL IHC (staining intensity)	Mean	0.7	0.7	13	0	0.939	0.3	0.8	21	0.5	0.010	0.099
	Range	[0, 2]	[0, 2]				[0, 1]	[0, 2]				
SNAIL mRNA (fold-change)	Mean	3.0	6.3	13	3.1	0.133	3.1	5.7	21	3.0	0.017	0.763
	Range	[-6.4, 9.7]	[2.4, 8.8]				[-10.8, 13.06]	[-4.5, 9.3]				
E-cadherin (staining intensity)	Mean	1.6	1.4	14	-0.2	0.443	1.9	1.9	22	0.0	0.987	0.538
	Range	[0.5, 2.5]	[0.5, 2.5]				[0.5, 3]	[0.5, 3.0]				
Clev Caspase-3 (% positive cells)	Mean	5.9	5.7	12	-0.2	0.875	5.6	5.5	20	0.1	0.940	0.953
	Range	[4, 9]	[3, 9]				[2.5, 10.8]	[2.3, 9.8]				
Ki67 (positive/1000 cells)	Mean	54.5	50.9	11	-3.6	0.449	52.9	54.1	17	1.2	0.355	0.323
	Range	[40, 83]	[36, 65]				[44.2, 63.3]	[45.7, 66.9]				

Rectal biopsy sections collected from patients before and after treatments were subjected to IHC and/or mRNA analysis to determine the expression of cellular biomarkers including EGFR, Ki-67, Snail, Cleaved Caspase-3 and E-cadherin, all of which have previously been reported to be modulated by PEG in cell culture and animal models. For IHC studies, 2–3 rectal biopsies were formalin fixed, paraffin embedded, sectioned and subjected to separate immunostainings. A semi- quantitative scale was used to evaluate immunoreactivity of epithelial cell and the extent of staining was graded and scored as 0 (negative staining); 1+ (10% stained cells), 2+ (10–50% stained cells), and 3+ (50% stained cells). EGFR protein expression analysis was also done using ELISA as described in the “Methods” section. In addition to the protein, we also studied the effect of PEG-8000 on the mRNA expression of EGFR and Snail. Freshly isolated rectal biopsies (1–2) from control and PEG treated subjects were subjected to RT-PCR for mRNA expression of EGFR and Snail. As shown no significant reduction in the immunohistochemical expression of EGFR was observed in the rectal biopsies from subjects after PEG treatment. The data from ELISA measurements also did not show any reduction in the protein expression of EGFR. No statistical difference was observed in proliferation marker Ki67 and apoptosis marker Cleaved Caspase 3 after PEG treatment. Similarly no changes were found in the expression of Snail and E-cadherin biomarkers after PEG treatment. To further asses if PEG may be altering EGFR and or Snail at the mRNA level, we performed by RT-PCR as describe in the Methods. As shown, the data was ambiguous as the mRNA levels were higher in both treated and untreated groups after PEG therapy.

^a^ significance of within-group change.

^b^ significance of between group change.

### Effect of PEG treatment on the expression epidermal growth factor receptor (EGFR)

Our preclinical studies have previously shown that EGFR downregulation via internalization and degradation through the ubiquitin-proteosomal pathway was integral to the anti-neoplastic efficacy of PEG [14]. This led us to posit that EGFR downregulation would be a robust biomarker for anti-neoplastic activity of PEG in this clinical trial. However, as shown in Table 2 and S1 Table, there was no significant reduction in the immunohistochemical expression of EGFR in the rectal biopsies from subjects after PEG treatment. To further explore the potential role of EGFR, we performed direct EGFR protein quantification in freshly frozen samples by ELISA as described in the Methods. The data from ELISA measurements (Table 2 and S1 Table) also did not show any reduction in the protein expression of EGFR. This may reflect several possibilities including relatively higher doses (up to 10% PEG) used in the preclinical studies compared to the lower doses used in this clinical studies; it is also possible that the duration of therapy was too short.

### Effect of PEG treatment on the expression of cellular proliferation

PEG has previously been shown to suppress colonic epithelial proliferation in both cell culture and the AOM-treated rat model as measured by Ki67 expression in animal models or WST-1 expression in cell culture [11]. As described in the Methods section, rectal biopsies were processed to determine the immunohistochemical expression of several biomarkers including well recognized proliferation marker Ki67. However, as shown in Table 2 and S1 Table, our data showed very little change in the PEG treated groups. Our laboratory has previously also shown that the antiproliferative effect of PEG was accompanied by marked suppression of SNAIL, leading to induction of E-cadherin and subsequent sequestration of β-catenin away from the nucleus [14]. Since inhibition of SNAIL signaling by PEG was found to be critical for its protective effect in preclinical settings, we performed immunohistochemical analyses to determine if the expression of SNAIL and E-cadherin was altered before and after PEG treatment. As shown in Table 2 and S1 Table, we again did not see any statistical difference in the expression of these biomarkers after PEG treatment. Even though the expression of transcriptional regulator SNAIL surprisingly was found to be higher in PEG treated subjects, it was offset by no change in the expression of E-cadherin.

### Effect of PEG treatment on the mRNA expression of EGFR and SNAIL

To further asses if PEG may be altering EGFR and or SNAIL at the message level, we performed mRNA determination by RT-PCR as describe in the Methods. However, the data were incongruous as the mRNA levels were higher in both treated and untreated groups after 6 months of therapy.

### Safety results

The frequency of Treatment Emergent Adverse Events (TEAEs) are shown in Table 3. TA review of the safety data indicated that GI TEAEs were the most common as expected; there was even a doubling in drug-related (possibly/probably/definitely) GI TEAEs in the 17 g vs. 8 g vs. placebo (48% vs. 23% vs. 17%) groups. The majority was diarrhea with flatulence and bloating including those scored grade 3.

**Table 3:** Adverse event frequencies.

	Placebo	PEG 3350 8 g	PEG 3350 17 g
	(n = 24)	(n = 26)	(n = 23)
# (%) of subjects with TEAEs	14 (58%)	15 (58%)	13 (57%)
# (%) of subjects with Grade 1 TEAEs	12 (50%)	12 (46%)	8 (35%)
# (%) of subjects with Grade 2 TEAEs	7 (29%)	7 (27%)	8 (35%)
# (%) of subjects with Grade 3 TEAEs	2 (8%)	3 (12%)	3 (13%)
# (%) subjects off study	0 (0%)	2(8%)	2 (9%)

## Discussion

We have performed the first randomized, double-blinded placebo controlled intervention trial in subjects at high risk for colorectal cancer to test the cancer prevention potential of polyethylene glycol, a widely available, safe, and inexpensive agent. Our goal was to assess the impact of two doses of PEG treatment for six months on biomarkers of CRC risk. We observed a trend that PEG supplementation decreased ACF, albeit it did not achieve statistical significance. On the other hand, other surrogate biomarkers especially related to mechanism (EGFR expression, proliferation, apoptosis etc.) showed no trend towards beneficial change making the overall message of this trial ambiguous. This may be related, in part, to the problems in execution of the study (patient attrition, sample retrieval issues etc.).

To our knowledge the only previously published data showed that PEG decreased adenoma occurrence in a case control study. While the effect size was promising, the fact that short term exposure (<30 days) appeared to confer marked clinical benefits seemed somewhat suspect from a biological plausibility perspective [17]. Our data establishes the safety of PEG supplementation for patients not with chronic constipation with no significant differences in adverse events (grade 1, 2 or 3) although there were 2 dropouts in each of the PEG treated groups (related to diarrhea). Indeed, prior to our study, there was very little data to assess the tolerability of PEG in non-constipated patients [17]. Thus, PEG may be a safe option for cancer prevention.

The timing of this trial is particularly apropos given that the field of chemoprevention has recently been reinvigorated with the USPSTF recommendation of aspirin for primary CRC (in conjunction with cardiovascular benefit) [7]. However, aspirin toxicity is still significant and if evaluated purely from a CRC perspective, the harms of aspirin may outweigh the benefit (6). COX2 inhibitors mitigate much of the GI toxicity of aspirin and have demonstrated striking efficacy in large scale randomized-controlled trial [22, 23] however unexpected cardiovascular toxicity makes this an unacceptable strategy [24, 25]. While there are no human studies comparing PEG and aspirin/NSAIDS, in animal models PEG appears to outperform these other agents [26].

In this trial, we observed a potential reduction of aberrant crypt foci (ACF) post PEG treatment (which failed to achieve statistical significance). ACF have been widely used in rodent models of chemoprevention. Furthermore, a landmark report by Takayama demonstrated that rectal ACFs were not only a risk marker in human colon carcinogenesis but also correlated with chemoprevention by sulindac [19]. Using similar chromoendoscopy techniques, we were able to detect decreases in rectal ACF from patients treated with PEG. However, given the lack of statistical significance, these differences should be interpreted cautiously. It is also noted that the difference was largely due to an increase in the placebo group with a slight decrease in the treated group. Since there may be potential subjectivity of ACF analysis, observers were blinded to the treatment group and all attempts were made to have the same endoscopist assay ACF in a particular patient at both the initial and final time-points, pre and post study. Our data, although suggestive of an ACF-lowering effect of PEG, did not reach formal statistical significance and thus needs corroboration. The fact that the base-line value of ACF in the placebo group was lower than in the treated groups (2.5 versus 6.0 and 4.6, respectively) illustrates the challenges of small, biomarker-based trials. Moreover, we should also acknowledge that several studies have impugned the robustness of the endoscopic detection of rectal ACF for both risk stratification and chemoprevention [20, 27, 28].

For this trial, we also measured a marker of cellular proliferation (Ki67) in the rectal mucosa. Rectal proliferation is well established marker of neoplasia elsewhere in the colon (field carcinogenesis) [29]. A number of previous preclinical studies have used these as intermediate biomarkers of chemoprevention although the reliability has been inconsistent [30–32]. Thus, it is not surprising to see no effect. Furthermore, recent data suggests that there may be changes in proliferation with even short term PEG (i.e. PEG purge prior to a colonoscopy) thus providing another potential confounder [33].

Whether PEG could have its anti-neoplastic effects as a result of direct effects on colonic mucosa or due to its effect on bowel motility (considering a link between constipation and CRC) is still an unresolved matter [34]. One of the important biological underpinnings of colon carcinogenesis is related to the mutagenic effects of major secondary bile acids such as deoxycholate [35, 36]. Deoxycholate levels are increased with increased colon transit time (constipation) and this would be coupled with increased mucosal contact time of these pro-neoplastic bile acids [37]. Indeed, by attenuating the effects of these pro-carcinogenic bile salts by other bile salts such as ursodeoxycholic acid, it has been shown to be protective in animals [38] and potentially in humans [39]. However, there are several other studies which do not support the role of constipation for increased risk in CRC [40–42]. Importantly, in rodent models it is reported that only PEG and not other cathartics, manifested a protective effect against colon carcinogenesis [43]. The fact that PEG works in cell culture would also argue for its more direct effect rather than just related to anti-constipation activities.

From a mechanistic perspective, we had previously developed biological model of PEG action focusing on EGFR downregulation [14]. EGFR, a transmembrane glycoprotein, is overexpressed early in colon carcinogenesis making it an excellent therapeutic candidate [44]. Indeed, studies have suggested that EGFR normalization may be a promising target for chemoprevention [45] including having an important role in aspirin efficacy [46]. Our laboratory has previously shown that PEG downregulates EGFR in both AOM-treated rat and human colon cancer cells, HT-29. This downregulation appears to be a result of increased endocytic lysosomal degradation. Moreover, using a ShRNA approach, we demonstrate that EGFR downregulation is central to PEG responsiveness with regards to its role in anti-proliferation. Based on these results, we tested if EGFR expression was reduced in subjects after PEG treatment. We had shown that this led to suppression of transcriptional regulator SNAIL triggering induction of E-cadherin [14]. This in turn sequestrates β-catenin to the plasma membrane and away from nuclear compartment to help decrease Wnt-driven signaling [14]. We had shown that this EGFR downregulation was also a hallmark of the suppression of head and neck carcinogenesis with topical PEG [47]. Therefore, based on these lines of evidence we had made EGFR levels our revised primary endpoint for the study. This was evaluated both at the level of message (mRNA) and protein (by two separate procedures of immunohistochemistry and ELISA). Surprisingly, there was no evidence of EGFR downregulation with PEG. Whether this means that PEG had no efficacy or PEG in humans utilize a different mechanism of action remains unclear. There may be other heretofore unexplored modalities such as modulation of microbiome [48–50], plasma membrane effects, different signal transduction pathways etc. that may potentially serve as better biomarkers for the putative anti-neoplastic effect of PEG in future studies [48, 49].

The strengths of the study relate to its double blinded, placebo-controlled randomized design, supported by robust pre-clinical data, and the novelty of testing PEG in a non-constipated, high-risk population (defined by a personal history of colorectal adenomas). From a clinical perspective, given that ~20% of the adult population is considered constipated, one may make the argument that a potential anti-neoplastic benefit may impact the cathartic choice. This may have large public health implications given the ubiquitous use of over-the-counter laxatives including PEG. On the other hand, there are also significant limitations that need to be acknowledged. The selection of endpoints in such trials is challenging, and we had to change our primary endpoint once it became clear that the entry criterion of 5 ACF was proving to be a recruitment barrier. The study is underpowered partly because sample size estimates were based on effect sizes seen in animal models. Indeed, we estimate that our actual sample size would yield the power to detect a statistically significant difference in ACF between placebo and 8g or 17g of PEG of only 16 and 46%, respectively. While recruitment was excellent, the significant missing data marred the trial. The lack of assessment of family history is a weakness of the study design. Furthermore, our primary outcome (EGFR downregulation) is based on mechanistic studies for preclinical models which may not translate into human studies [14].

In conclusion, this trial is the first in human trial to address the role of PEG in prevention of human colonic neoplasia. While imperfect from a variety of perspectives (limitations of individual biomarkers, missing data etc.), this was a rigorously designed trial and gives some insight into safety and efficacy of PEG. Although the primary endpoint was negative, the trend towards ACF reduction is somewhat provocative. One would consider that if there can be other support from epidemiological studies, PEG would warrant a larger scale and longer term adenoma prevention trial or utilization of more robust biomarkers for chemoprevention such as spectral markers which were validated in a recent Phase 2B chemoprevention trial with aspirin [50]. The more general caveat for future studies is that mechanistic insights from preclinical models may not translate into reliable intermediate biomarkers in clinical studies [48].

## Supporting information

S1 CONSORT Checklist CONSORT checklist. (DOC)

S1 Table Supplemental Table 1. (DOCX)

S2 Table Supplemental Table 2. (XLSX)

S1 Text Study protocol. (DOC)
