# Supplementary material for: Prevention of colonic neoplasia with polyethylene glycol: A short term randomized placebo-controlled double-blinded trial
Source: PLoS One. 2018 Apr 4;13(4):e0193544. doi: 10.1371/journal.pone.0193544 (PMC5884487; doi:10.1371/journal.pone.0193544)
Supplement: S1 Table — (DOCX) [file pone.0193544.s002.docx]

**Table 1. Demographics of study subjects.**

|  | Placebo | PEG 8 g daily | PEG 17 g daily |
| --- | --- | --- | --- |
|  | (*n* = 24) | (*n* = 27) | (*n* = 24) |
| Age, years (mean, range) | 59.0 (36 - 74) | 63.4 (46 - 80) | 58.7 (30 - 72) |
| Male | 13 (54.2%) | 13 (48.1%) | 12 (50.0%) |
| Race: Asian | 2 (8.3%) | 0 (0.0%) | 0 (0.0%) |
| Race: Black or African American | 0 (0.0%) | 4 (14.8%) | 4 (16.7%) |
| Race: White | 22 (91.7%) | 23 (85.2%) | 20 (8.3%) |
| Ethnicity: Hispanic or Latino | 0 (0.0%) | 2 (7.4%) | 1 (4.2%) |
| Smoking Status: Current | 1 (4.2%) | 0 (0.0%) | 4 (16.7%) |
| Smoking Status: Past | 11 (45.8%) | 15 (55.6%) | 11 (45.8%) |
| Smoking Status: Unknown | 0 (0.0%) | 0 (0.0%) | 1 (4.2%) |
| BMI (mean, range) | 28.1 (19.5 - 47.0) | 28.5 (20.4 - 49.4) | 29.5 (21.7 - 44.2) |
| Current use of NSAIDs: Yes | 4 (16.7%) | 6 (22.2%) | 1 (4.2%) |
| Current use of NSAIDs: Unknown | 0 (0.0%) | 0 (0.0%) | 1 (4.2%) |

**Table 2. Changes in mean values of biomarkers in treated and placebo groups, with high and low PEG doses combined.**

|  | | Control (Placebo) | | | | | Intervention (8 g or 17 g PEG) | | | | | |
| --- | --- | --- | --- | --- | --- | --- | --- | --- | --- | --- | --- | --- |
|  |  | Pre-treatment | Post-treatment | n | change | p1^a^ | Pre-treatment | Post-treatment | n | change | p1^a^ | p2^b^ |
| Rectal ACF (number/rectum) | Mean | 2.5 | 4.2 | 13 | 1.7 | 0.148 | 5.4 | 5.1 | 19 | 0.3 | 0.598 | 0.109 |
|  | Range | [0, 13] | [0, 11] |  |  |  | [0, 9] | [0, 16] |  |  |  |  |
| EGFR IHC (staining intensity) | Mean | 1.93 | 2.14 | 14 | 0.2 | 0.256 | 2.0 | 1.9 | 22 | 0.1 | 0.621 | 0.280 |
|  | Range | [1, 3] | [1, 3] |  |  |  | [1, 3] | [0, 3] |  |  |  |  |
| EGFR mRNA (fold-change) | Mean | 1.76 | 5.04 | 13 | 2.7 | 0.023 | 1.8 | 3.9 | 21 | 2.1 | 0.054 | 0.620 |
|  | Range | [-6.27, 7.18] | [0.05, 7.17] |  |  |  | [-8.28, 7.56] | [-8.4, 8.4] |  |  |  |  |
| EGFR ELISA (ng/100g protein) | Mean | 3.09 | 3.75 | 6 | 0.7 | 0.173 | 3.9 | 4.0 | 10 | 0.1 | 0.721 | 0.448 |
|  | Range | [0.4, 6.4] | [2.2, 6.6] |  |  |  | [-0.48, 7.29] | [0.1, 8.3] |  |  |  |  |
| SNAIL IHC (staining intensity) | Mean | 0.7 | 0.7 | 13 | 0 | 0.939 | 0.3 | 0.8 | 21 | 0.5 | 0.010 | 0.099 |
|  | Range | [0, 2] | [0, 2] |  |  |  | [0, 1] | [0, 2] |  |  |  |  |
| SNAIL mRNA (fold-change) | Mean | 3.0 | 6.3 | 13 | 3.1 | 0.133 | 3.1 | 5.7 | 21 | 3.0 | 0.017 | 0.763 |
|  | Range | [-6.4, 9.7] | [2.4, 8.8] |  |  |  | [-10.8, 13.06] | [-4.5, 9.3] |  |  |  |  |
| E-cadherin (staining intensity) | Mean | 1.6 | 1.4 | 14 | -0.2 | 0.443 | 1.9 | 1.9 | 22 | 0.0 | 0.987 | 0.538 |
|  | Range | [0.5, 2.5] | [0.5, 2.5] |  |  |  | [0.5, 3] | [0.5, 3.0] |  |  |  |  |
| Clev Caspase-3 (% positive cells) | Mean | 5.9 | 5.7 | 12 | -0.2 | 0.875 | 5.6 | 5.5 | 20 | 0.1 | 0.940 | 0.953 |
|  | Range | [4, 9] | [3, 9] |  |  |  | [2.5, 10.8] | [2.3, 9.8] |  |  |  |  |
| Ki67 (positive/1000 cells) | Mean | 54.5 | 50.9 | 11 | -3.6 | 0.449 | 52.9 | 54.1 | 17 | 1.2 | 0.355 | 0.323 |
|  | Range | [40, 83] | [36, 65] |  |  |  | [44.2, 63.3] | [45.7, 66.9] |  |  |  |  |

Table 2: Effect of PEG treatment on the rectal mucosal expression of biomarker panel previously validated in pre-clinical studies. Rectal biopsy sections collected from patients before and after treatments were subjected to IHC and/or mRNA analysis to determine the expression of cellular biomarkers including EGFR, Ki-67, Snail, Cleaved Caspase-3 and E-cadherin, all of which have previously been reported to be modulated by PEG in cell culture and animal models. For IHC studies, 2-3 rectal biopsies were formalin fixed, paraffin embedded, sectioned and subjected to separate immunostainings. A semi- quantitative scale was used to evaluate immunoreactivity of epithelial cell and the extent of staining was graded and scored as 0 (negative staining); 1+ (10% stained cells), 2+ (10-50% stained cells), and 3+ (50% stained cells). EGFR protein expression analysis was also done using ELISA as described in the “Methods” section. In addition to the protein, we also studied the effect of PEG-8000 on the mRNA expression of EGFR and Snail. Freshly isolated rectal biopsies (1-2) from control and PEG treated subjects were subjected to RT-PCR for mRNA expression of EGFR and Snail. As shown no significant reduction in the immunohistochemical expression of EGFR was observed in the rectal biopsies from subjects after PEG treatment. The data from ELISA measurements also did not show any reduction in the protein expression of EGFR. No statistical difference was observed in proliferation marker Ki67 and apoptosis marker Cleaved Caspase 3 after PEG treatment. Similarly no changes were found in the expression of Snail and E-cadherin biomarkers after PEG treatment. To further asses if PEG may be altering EGFR and or Snail at the mRNA level, we performed by RT-PCR as describe in the Methods. As shown, the data was ambiguous as the mRNA levels were higher in both treated and untreated groups after PEG therapy.

^a^ significance of within-group change.

^b^ significance of between group change.

**Table 3. Adverse event frequencies**

|  | Placebo | PEG 3350 8 g | PEG 3350 17 g |
| --- | --- | --- | --- |
|  | (n = 24) | (n = 26) | (n = 23) |
| # (%) of subjects with TEAEs | 14 (58%) | 15 (58%) | 13 (57%) |
| # (%) of subjects with Grade 1 TEAEs | 12 (50%) | 12 (46%) | 8 (35%) |
| # (%) of subjects with Grade 2 TEAEs | 7 (29%) | 7 (27%) | 8 (35%) |
| # (%) of subjects with Grade 3 TEAEs | 2 (8%) | 3 (12%) | 3 (13%) |
| # (%) subjects off study | 0 (0%) | 2(8%) | 2 (9%) |
