# Supplementary material for: Prevention of colonic neoplasia with polyethylene glycol: A short term randomized placebo-controlled double-blinded trial
Source: PLoS One. 2018 Apr 4;13(4):e0193544. doi: 10.1371/journal.pone.0193544 (PMC5884487; doi:10.1371/journal.pone.0193544)
Supplement: S1 Text — (DOC) [file pone.0193544.s004.doc]

**DCP Protocol #:** NWU06-8-01

**Local Protocol #:** NCI06-8-01

**POLYETHYLENE GLYCOL FOR ACF REDUCTION AND BIOMARKER MODULATION IN INDIVIDUALS WITH CRC RISK**

**Consortium Name:** The National Cancer Chemoprevention Group

**Name of Consortium Principal** Seema A. Khan, MD

**Investigator:** 303 E. Superior, Suite 4-111

Chicago, IL 60611

Tel: 312-503- 4236

Fax: 312-503-2555

skhan@nm.org

**Protocol Chair:** Hemant K. Roy, MD

Boston Medical Center

EBRC, 5^th^ floor, room 527

650 Albany St.

Boston, MA 02118

Tel: 617-638-8330

Fax: 617-638-7785

**Protocol Lead Investigator at** Hemant Roy

**Boston University:** Boston Medical Center

EBRC, 5th floor, room 527

650 Albany Street

Boston, MA 02118

Tel: 617-638-8330

Fax: 617- 638-7785

Hemant.Roy@bmc.org

**Protocol Lead Investigator at** Sonia Kupfer, MD

**University of Chicago:** University of Chicago

Section of Gastroenterology

900 East 57th Street, MB#9

Chicago, IL 60637

773-702-8076 (phone)

773-702-2281 (fax)

[skupfer@medicine.bsd.uchicago.edu](mailbox://C|/Users/aad499/AppData/Roaming/Thunderbird/Profiles/vdng4y5h.default/Mail/hecky.it.northwestern.edu/skupfer@medicine.bsd.uchicago.edu)

**Co-Investigator at** David T. Rubin, MD

**University of Chicago:** University of Chicago

Section of Gastroenterology

5841 S. Maryland Ave., MC 4080

Chicago, IL 60637

Tel: 773-702-4708

Fax: 773-702-5790

drubin@medicine.bsd.uchicago.edu

**Protocol Lead Investigator at** Laura Bianchi

**NorthShore University HealthSystem:**Northshore University HealthSystem

2650 Ridge Avenue G221

Evanston, IL 60201

Tel: 847-657-1900

Fax: 847-733-5407

[lbianchi@northshore.org](mailto:lbianchi@northshore.org)

**Statistician:** Borko D. Jovanovic, Ph.D.

Associate Professor

Department of Preventive Medicine

The Feinberg School of Medicine, Northwestern University

680 North Lake Shore Drive, Suite 1102

Tel: 312.503.2008

Fax: 312- 908-9588

[borko@northwestern.edu](mailto:borko@northwestern.edu)

**Sponsor:** NCI/Division of Cancer Prevention

6130 Executive Blvd., Rm 2117

Bethesda, MD 20892 (For FedEx, use Rockville, MD 20852)

(301) 496-8563

**IND# 103,600**

**Agent(s)/Supplier**: Polyethylene Glycol 3350 (PEG 3350)

NorthShore University HealthSystem Pharmacy

**NCI Contract #**  N01-CN-35157

**Protocol Version Date:** January 14, 2015

**Protocol Revision or**

**Amendment #** Version 4.6

**DCP Monitoring** Westat

**Contractor:** 1441 W. Montgomery Ave.

Rockville, MD 20850

**SCHEMA**

Polyethylene Glycol for Chemoprevention of Colon Carcinogenesis

**Flex Sig**

**5 rectal bx**

**n=20**

**PEG 3350 8g/day n=35**

**105 Subjects w/ history of neoplasia**

**140 Subjects undergoing colonoscopy for h/o neoplasia**

**PEG 3350 17g/day**

**n=35**

**Placebo**

**n=35**

**Rectal ACF Analysis**

**Biopsy**

**Randomize**

**Flex Sig**

**5 rectal bx**

**n=20**

**Flex Sig**

**5 rectal bx**

**n=20**

**Treat for 6 months**

**~10 pt per group drop out**

Figure 1

**TABLE OF CONTENTS**

[**SCHEMA iii**](#__RefHeading___Toc179017537)

[**TABLE OF CONTENTS** iii](#__RefHeading___Toc179017538)

[**TABLE OF CONTENTS** iv](#__RefHeading___Toc179017539)

[**1.** **OBJECTIVES** 1](#__RefHeading___Toc179017540)

[1.1 Primary Objective 1](#__RefHeading___Toc179017541)

[1.2 Secondary Objective 1](#__RefHeading___Toc179017542)

[**2.** **BACKGROUND** 1](#__RefHeading___Toc179017543)

[2.1 Colorectal Cancer 1](#__RefHeading___Toc179017544)

[2.2 Polyethylene Glycol 3350 1](#__RefHeading___Toc179017545)

[2.3 Rationale 2](#__RefHeading___Toc179017546)

[**3.** **SUMMARY OF STUDY PLAN** 8](#__RefHeading___Toc179017547)

[**4.** **PARTICIPANT SELECTION** 10](#__RefHeading___Toc179017548)

[4.1 Inclusion Criteria 10](#__RefHeading___Toc179017549)

[4.2 Exclusion Criteria 11](#__RefHeading___Toc179017550)

[4.3 Inclusion of Women and Minorities 12](#__RefHeading___Toc179017551)

[4.4 Recruitment and Retention Plan 12](#__RefHeading___Toc179017552)

[**5.** **AGENT ADMINISTRATION** 12](#__RefHeading___Toc179017553)

[5.1 Dose Regimen and Dose Groups 12](#__RefHeading___Toc179017554)

[5.2 PEG 3350 Administration 12](#__RefHeading___Toc179017555)

[5.3 Run-in Procedures 12](#__RefHeading___Toc179017556)

[5.4 Contraindications 13](#__RefHeading___Toc179017557)

[5.5 Concomitant Medications 13](#__RefHeading___Toc179017558)

[5.6 Dose Modification 13](#__RefHeading___Toc179017559)

[5.7 Adherence/Compliance 14](#__RefHeading___Toc179017560)

[**6.** **PHARMACEUTICAL INFORMATION** 14](#__RefHeading___Toc179017561)

[6.1 Polyethylene Glycol 3350 14](#__RefHeading___Toc179017562)

[6.2 Reported Adverse Events and Potential Risks 14](#__RefHeading___Toc179017563)

[6.3 Availability 15](#__RefHeading___Toc179017564)

[6.4 Agent Distribution 15](#__RefHeading___Toc179017565)

[6.5 Agent Accountability 15](#__RefHeading___Toc179017566)

[6.6 Packaging and Labels 15](#__RefHeading___Toc179017567)

[6.7 Storage 15](#__RefHeading___Toc179017568)

[6.8 Registration/Randomization 16](#__RefHeading___Toc179017569)

[6.9 Blinding and Unblinding Methods 16](#__RefHeading___Toc179017570)

[6.10 Agent Destruction/Disposal 16](#__RefHeading___Toc179017571)

[**7. CLINICAL EVALUATIONS AND PROCEDURES** 17](#__RefHeading___Toc179017572)

[7.1 Schedule of Events 17](#__RefHeading___Toc179017573)

[7.2 Baseline Testing/Pre-study Evaluation 17](#__RefHeading___Toc179017574)

[7.3 Evaluations During Study Intervention 18](#__RefHeading___Toc179017575)

[7.4 Evaluations at Completion of Study Intervention 19](#__RefHeading___Toc179017576)

[7.5 Post-intervention Follow-up Period 19](#__RefHeading___Toc179017577)

[7.6 Methods for Clinical Procedures 19](#__RefHeading___Toc179017578)

[**8.** **CRITERIA FOR EVALUATION AND ENDPOINT DEFINITION** 21](#__RefHeading___Toc179017579)

[8.1 Primary Endpoint 21](#__RefHeading___Toc179017580)

[8.2 Secondary Endpoints 21](#__RefHeading___Toc179017581)

[8.3 Off Agent Criteria 21](#__RefHeading___Toc179017582)

[8.4 Off Study Criteria 21](#__RefHeading___Toc179017583)

[8.5 Study Termination 21](#__RefHeading___Toc179017584)

[9.1 Rationale for Methodology Selection 21](#__RefHeading___Toc179017585)

[9.2 Comparable Methods 22](#__RefHeading___Toc179017586)

[**10.** **SPECIMEN MANAGEMENT** 22](#__RefHeading___Toc179017587)

[10.1 Laboratories 22](#__RefHeading___Toc179017588)

[10.2 Collection and Handling Procedures 23](#__RefHeading___Toc179017589)

[10.3 Shipping instructions 23](#__RefHeading___Toc179017590)

[10.4 Tissue Banking 23](#__RefHeading___Toc179017591)

[**11.** **REPORTING ADVERSE EVENTS** 23](#__RefHeading___Toc179017592)

[11.1 Adverse Events 23](#__RefHeading___Toc179017593)

[11.2 Serious Adverse Events 24](#__RefHeading___Toc179017594)

[**12.** **STUDY MONITORING** 26](#__RefHeading___Toc179017595)

[12.1 Data Management 26](#__RefHeading___Toc179017596)

[12.2 Case Report Forms 26](#__RefHeading___Toc179017597)

[12.3 Source Documents 26](#__RefHeading___Toc179017598)

[12.4 Data and Safety Monitoring Plan 26](#__RefHeading___Toc179017599)

[12.5 Sponsor or FDA Monitoring 26](#__RefHeading___Toc179017600)

[12.6 Record retention 27](#__RefHeading___Toc179017601)

[12.7 Cooperative Research and Development Agreement (CRADA)/Clinical Trials Agreement (CTA) 27](#__RefHeading___Toc179017602)

[**13.** **STATISTICAL CONSIDERATIONS** 27](#__RefHeading___Toc179017603)

[13.1 Study Design/Endpoints 27](#__RefHeading___Toc179017604)

[13.2 Sample Size/Accrual Rate 27](#__RefHeading___Toc179017605)

[13.3 Randomization and Stratification 28](#__RefHeading___Toc179017606)

[13.4 Primary Endpoint(s) 28](#__RefHeading___Toc179017607)

[13.5 Secondary Endpoint(s) 28](#__RefHeading___Toc179017608)

[13.6 Reporting and Exclusions 28](#__RefHeading___Toc179017609)

[13.7 Evaluation of Toxicity 28](#__RefHeading___Toc179017610)

[13.8 Evaluation of Response 28](#__RefHeading___Toc179017611)

[13.9 Interim Analysis 29](#__RefHeading___Toc179017612)

[13.10 Ancillary Studies 29](#__RefHeading___Toc179017613)

[**14.** **ETHICAL AND REGULATORY CONSIDERATIONS** 29](#__RefHeading___Toc179017614)

[14.1 Form FDA 1572 29](#__RefHeading___Toc179017615)

[14.2 Other Required Documents 29](#__RefHeading___Toc179017616)

[14.3 Institutional Review Board Approval 29](#__RefHeading___Toc179017617)

[14.4 Informed Consent 29](#__RefHeading___Toc179017618)

[14.5 Submission of Regulatory Documents 30](#__RefHeading___Toc179017619)

[14.6 Other 30](#__RefHeading___Toc179017620)

[**15.** **Financing, Expenses, and/or Insurance** 30](#__RefHeading___Toc179017621)

[**CONSENT FORM** 31](#__RefHeading___Toc179017622)

[APPENDIX A - Performance Status Criteria A](#__RefHeading___Toc179017623)

[**Appendix B - Patient Diary** B](#__RefHeading___Toc179017624)

[**Appendix C - Victoria Bowel Performance Scale** C](#__RefHeading___Toc179017625)

[**REFERENCES** D](#__RefHeading___Toc179017626)

1. **OBJECTIVES**
   1. **Primary Objective**

The primary objective of this study is to evaluate the effect of polyethylene glycol (PEG) 3350 (administered at 8g or 17g/day for six months) versus placebo on EGFR expression. If either dose of PEG treatment results in a significant reduction in EGFR expression, the trial will be considered a success.

**1.2 Secondary Objective**

The secondary objectives (in order of priority) are

- To determine the effect of PEG 3350 on aberrant crypt foci (ACF) number and to compare the reduction in ACF number between the low dose (8g PEG 3350 / day) and higher dose (17g PEG 3350 / day) groups.
- To determine the effect of PEG 3350 on mucosal epithelial proliferation (Ki-67)
- To determine the effect of PEG 3350 on mucosal apoptosis (cleaved caspase-3)
- To determine the effect of PEG 3350 on SNAIL protein expression
- To determine the effect of PEG 3350 on mRNA expression of SNAIL and EGFR.

**2. BACKGROUND**

**2.1** **Colorectal Cancer**

Colorectal cancer (CRC) remains the second leading cause of cancer-related mortality in the USA(3), with an estimated 153,760 new cases and 52,180 deaths from this disease in 2007. The lifetime risk for an American developing CRC is 6%.(4) CRC is curable if discovered at an early, localized stage. However, early stage CRC is insidious and generally asymptomatic. The classic symptoms of hematochezia, anemia, altered bowel habits, abdominal pain and weight loss are typically not seen until advanced, and thus less curable, stages of the disease.

In order to address the fatality rate of CRC, efforts are aimed at screening asymptomatic at-risk (age >50 years) populations. There have been a variety of techniques advocated for use in CRC screening, including fecal analysis for occult blood, DNA, radiological-based analysis (barium enema and CT colography), and endoscopic-based analysis (flexible sigmoidoscopy and colonoscopy).*(5,* 6) Many of these techniques have been rigorously demonstrated to decrease both morbidity and mortality. Despite the array of screening options available, the majority of the population does not receive any CRC screening due to patient reluctance, discomfort, embarrassment, cost, and other factors.(7)

Chemoprevention represents a promising modality for decreasing CRC incidence and death rates. Numerous agents have been shown to be effective in experimental and case-control studies; however, many have failed in more rigorous prospective clinical trials. For instance, the epidemiological and experimental data that fiber is protective is unequivocal(8); yet a number of rigorously performed prospective double-blinded adenoma prevention trials have all failed to show efficacy.*(9,* 10) Similarly, several groups including ours have shown that ursodeoxycholic acid is a remarkably potent protective agent in the azoxymethane-treated rat model of experimental colon carcinogenesis.(11, 12) However, a recent prospective clinical trial showed no reduction in the number of total adenomas.(13)

.

**2.2** **Polyethylene Glycol 3350**

Studies with the azoxymethane (AOM)-treated rat model of colon carcinogenesis indicate that polyethylene glycol (PEG) is one of the most potent chemopreventive agents with respect to inhibiting colon carcinogenesis.(14-16) Moreover, PEG is well-tolerated and is available by prescription for treatment of constipation in the United States. There is minimal absorption of PEG, thereby minimizing untoward systemic effects. Indeed, its safety profile is underscored by its long history of over-the-counter status in several European countries, and also the recent approval of OTC status for Miralax (PEG 3350) in the United States (to be marketed by Schering-Plough).

PEG is widely used in the United States as a laxative. However, PEG may offer chemopreventive benefit without inducing long-term diarrhea. PEG’s efficacy at standard doses (17g of PEG 3350) is modest, causing < ½ bowel movement per day increase over placebo.(17) Clinical studies indicate a degree of tachyphylaxis to PEG’s stool-altering effects within 1 month.(18) Additionally, data from experimental models suggest that non-laxative doses are efficacious at preventing colon cancer. Finally, the observation that a number of non-PEG laxatives failed to offer CRC protection indicates that the chemopreventive effect of PEG does not appear to be directly related to its laxation ability.(19) Even if there is a slight increase in bowel movements, there may be little-to-no negative clinical impact. This is because 20-30% of the population is estimated to have clinical constipation, and thus are eligible for PEG treatment anyway.(20) Constipation worsens with age (as does CRC risk) and constipation has been implicated as a CRC risk factor. Taken together the data from preclinical models (e.g., AOM-treated rat, MIN mouse and cell culture) has been compelling and is supported by epidemiological studies. However, the clinical ability of PEG has not been rigorously assessed. Further epidemiological studies are limited by methodological flaws. A rigorous evaluation of PEG’s chemopreventive efficacy and side effect profile in humans is therefore needed.

**2.3** **Rationale**

2.3.1 Inadequacy of current chemopreventive agents

While there have been a number of agents that have demonstrated chemopreventive efficacy against colon cancer, clinical application has been limited by either: (A) toxicity and/or (B) sub-optimal efficacy.(21) PEG is not absorbed systemically and has an outstanding clinical safety profile. Furthermore, preclinical studies have consistently demonstrated that PEG is one of the most potent chemopreventive agents against CRC.(14) While there is some case-control evidence to suggest that PEG has efficacy, more rigorous confirmation of the clinical efficacy is required. This proposed trial is the first prospective randomized trial aimed at evaluating the chemopreventive efficacy of PEG, using intermediate biomarkers relevant to colon carcinogenesis.

2.3.2 Examples of chemopreventive agents that have failed due to toxicity

(i) Aspirin: Both case control and placebo randomized trials have shown a moderate 30-50% efficacy in preventing colonic neoplasia.(22, 23) However, chronic consumption of aspirin is associated with a high rate of GI-toxicity (ulceration, perforation and hemorrhages).(24, 25) In fact, NSAID-related gastrointestinal complications rank 15^th^ among the most common causes of death in the United States.(26)

(ii) Celecoxib: Even though the use of celecoxib (a selective COX-2 inhibitor with low GI-toxicity) has been shown to produce an approximately 60% reduction in advanced adenomas and an approximately 40% reduction in total adenomas, its use has also been linked to increased cardiovascular risk.(27-31) Similarly, another COX-2 inhibitor, rofecoxib, was also shown to be associated with increased cardiovascular risk among patients with colorectal adenomas.(32)

(iii) Exisulind: A variant NSAID with no luminal GI-toxicity, exisulind has chemopreventive efficacy, but also causes hepatotoxicity.(33)

(iv) Hormone Replacement Therapy (HRT): In a landmark study, by the Women’s Health Initiative, estrogens were prospectively shown to decrease the incidence of colon cancer by 37%. However, further analysis showed that estrogen use was associated with an increase risk of both cardiovascular deaths and breast cancer, thus negating any benefit from colon cancer chemoprevention.(34, 35)

2.3.3 Examples of Agents limited by efficacy

a) Statins: While statins may be better tolerated and offered a 50% CRC risk reduction in a case control trial, it is estimated that the number needed to treat (NNT) to prevent one CRC was 4814 subjects.(36) Furthermore, recent cohort studies have failed to show any efficacy of statins for CRC prevention.(37)

b) Fiber: Even though certain observational and epidemiological studies indicate chemopreventive efficacy, results from prospective trials have been disappointing. Fiber supplements, in the form of daily wheat bran or ispaghula husk, have failed to reduce the recurrence of colorectal adenomas in high-to moderate-risk patients.(38)


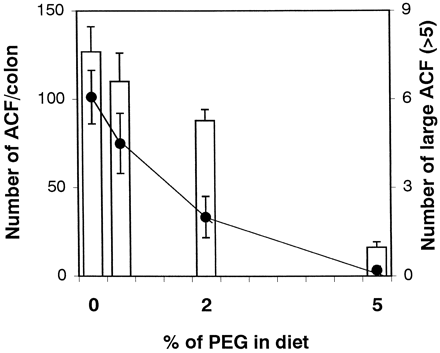


Figure 2 Potency of PEG for ACF reduction in the AOM-treated rat model (*Parnaud et al Can Res 1999*)

c) Others: Inverse associations between calcium intake and CRC have been identified. However adenoma intervention trials have been discordant, with meta-analysis showing at best equivocal efficacy.(39) Trials evaluating β-carotene alone, or in combination with vitamin E, reported no significant effect on recurrent adenomas.(40) While our lab was one of the pioneers of ursodeoxycholic acid, the results of clinical studies have been disappointing (no change in total adenomas, and only modest decreases in advanced adenomas).(13) In epidemiological studies folate has been widely shown to be associated with a lower risk of adenomas and carcinomas. However, a recent analysis of a randomized controlled trial suggests that folate may actually predispose to larger lesions. (41)

2.3.4 Clinical Safety of PEG

PEG is widely used in the United States as a laxative. While its efficacy at standard doses (17g of PEG 3350) is modest (<1/2 bowel movement per day increase over placebo), the safety profile has been outstanding.(17) Not only is PEG an inert molecule, but there is minimal, if any, systemic absorption. In several European countries, PEG is sold as an over-the-counter laxative (e.g. Forlax in France). Studies from animal models of CRC suggest that a sub-diarrheal dose may be effective for chemoprevention. (42) Even if there is a slight increase in bowel movements, there may be little-to-no negative clinical impact. This is because 20-30% of the population is estimated to have clinical constipation, and thus eligible for PEG treatment anyway.(20) Constipation worsens with age (as does CRC risk), and constipation has been implicated as a CRC risk factor.(20)

While PEG 3350 is indicated for short term treatment of constipation (approved for one week OTC and two weeks by prescription), the data on long term safety has been supported by a recent publication. (43) In an open label, single treatment, multi-center study, 311 patients received 17g of PEG 3350 for 12 months. In this study, 184 patients completed until Day 360. Of the dropouts, 23 dropped out for adverse events. The majority of these adverse events occurred within the first period with much less attrition at longer time points. In the population, 2.9% had abdominal distention, 10.6% had diarrhea, 7.4% had flatulence, 3.9% had loose stools and 5.5% had nausea. Importantly, no serious adverse event was considered related to study medication.

With regards to duration of treatment, studies in the AOM-treated rat indicate that ACF reduction by PEG is rapid (within several days of treatment).(44) There have been few chemopreventive studies in humans and no time course has been done. Shpitz and colleagues treated patients for >1 year and noted that low dose aspirin decreased ACF in patients. (45) Takayama noted a decrease in endoscopically-detected ACF with sulindac treatment for ~11 months. (46) More recently these authors performed a small open-label trial with sulindac and found that the number of ACF was reduced markedly in only 2 months. (2) Thus, while the ideal time point has not been elucidated, 6 months would appear to be a conservative estimate given the experimental and human studies on ACF reduction.

2.3.5 Experimental Studies:

The initial work of Pernaud and Corpet demonstrated the chemopreventive efficacy of PEG in the AOM-treated rat (80-95% suppression of ACF). [Figure 2]. (42) They demonstrated that PEG in the diet resulted in a dose-dependent suppression of ACF [Figure 2]. Studies from the Roy and Wali lab confirmed PEG’s efficacy in ACF and also tumors. (15, 16)To put this data in perspective, the Roy and Wali lab has previously published that NSAIDS resulted in a 33% reduction in ACF number.(47) Thus, the 95% suppression seen with PEG represents high activity. The Roy lab has also published pilot work in the MIN mouse, proving that the efficacy of PEG is not model specific.(15) Corpet recently catalogued the effectiveness of all published chemopreventive agents.(14) As indicated by [Figure 3], PEG outperformed conventional agents such as NSAIDS, vitamin D and ursodeoxycholic acid. Finally there is preliminary case-control data suggesting that PEG has clinical efficacy.(48) Together, these studies support the notion that PEG has great promise as an efficacious chemopreventive agent.

Figure 3 Mean Potency of some of the chemopreventive agents against colon Neoplasia. (Data modified from *Corpet and Tache, Nutn and Cancer 2002;* )

Cellular Mechanism of Action: Roy et al. has previously reported that PEG treatment caused a rapid and potent induction of apoptosis in two human colon cancer cell lines, CaCo-2 and HT-29.(49) They have confirmed this induction of apoptosis in the MIN mouse model of intestinal tumorigenesis.(15) Several groups have reported that PEG treatment can inhibit proliferation in a number of cell lines, including HT-29 cells and CaCo-2s.(50) Moreover, Roy et al. recently published that PEG treatment suppressed the epithelial hyperproliferation of early colon carcinogenesis in the AOM-treated rat.(16)

The Role of β-catenin: Given the critical role of β-catenin signaling in the initiation of colon carcinogenesis(51) and as a target for chemoprevention, Roy et al assessed signaling through the TOPFLASH reporter assay. In this assay, β-catenin activity is assessed by assaying the transcriptional activity of β-catenin’s downstream effector, Tcf. PEG treatment was found to be more potent than NSAIDS in suppressing Tcf signaling. Roy et al then investigated the mechanism through which PEG suppressed β-catenin signaling, and found that PEG did not alter β-catenin levels, but rather subcellular localization.(16) The data indicated that the sequestration of β-catenin to the plasma membrane (and hence away from the nucleus) occurred through interaction with the up-regulated E-cadherin. Overexpression of E-cadherin is an increasingly recognized mechanism of action of chemopreventive agents against colon cancer (e.g. refecoxib). (52) In order to elucidate how E-cadherin is induced by PEG treatment, Roy et al focused on its transcriptional repressor SNAIL. SNAIL has been implicated by our group and others as important in human colon carcinogenesis.(53, 54). Roy et al have demonstrated that selectively targeting SNAIL significantly suppressed tumor formation in the MIN mouse.(55) Roy et al found that SNAIL protein levels were markedly decreased by PEG treatment. Thus, the working paradigm for PEG chemopreventive activity is that PEG treatment → ↓ SNAIL protein → ↑E-cadherin → ↓ nuclear β-catenin → ↓ Tcf-regulated gene transcription → ↓ cell proliferation & ↑apoptosis.(16)

Figure 4. PEG treatment reverses AOM-induced EGFR Overexpression in the preneoplastic colonic mucosa

SNAIL regulation mechanism: The above model for PEG action, while compelling, does not address the mechanism through which PEG can regulate the intracellular protein, SNAIL. The bulky nature of PEG suggests that cell surface receptor interactions are likely. The epidermal growth factor receptor (EGFR) appeared to be a logical candidate given that it is important in colon carcinogenesis and known to regulate SNAIL levels.(56) Furthermore, targeting EGFR appears to be a potent chemopreventive approach.(57) The initial approach was to assess EGFR in the uninvolved mucosa of the AOM-treated rat. As can be seen in [Figure 4], induction of carcinogenesis (through AOM treatment) increased EGFR levels, and this was almost completely mitigated by PEG supplementation. A similar magnitude of reduction of EGFR protein, but not the mRNA expression (message), in the human colon cancer cell line HT-29 was also confirmed. This strongly suggests that PEG regulated EGFR through alteration in protein turnover, and not transcriptionally. Given the importance of the ubiquitin pathway in controlling EGFR protein levels, HT-29 cells were pre-treated with a lysosomal inhibitor and it was noted that this abrogated PEG-induced EGFR downregulation. While this data clearly demonstrates that PEG treatment induces EGFR internalization, and hence degradation, a fundamental issue is whether this is necessary for the chemopreventive efficacy of PEG. Therefore, HT-29 knockdowns for EGFR were constructed, using a stable Sh-RNA approach. As shown in [Figure 5] the EGFR knockdowns were much less sensitive to PEG-induced anti-proliferative effects, when compared to wild-type (empty-vector) cells.

Figure 5: Blunted response of PEG on proliferation (PCNA expression) in EGFR knockdown HT-29 cells*.*

Finally, in order to determine whether EGFR downregulation was responsible for the PEG-induced SNAIL decrease, Sh-RNA constructs were used. As expected, these constructs had less SNAIL expression detectable by flow cytometric analysis. Importantly, PEG treatment in these constructs failed to cause any further significant decrease in SNAIL levels, thereby implicating EGFR in the SNAIL deregulation [Figure 6].

Figure 6: FACS analysis: HT-29 cells were serum starved for 72h and treated with PEG for 24 h and then stained with either mouse monoclonal anti-EGFR (528), for membrane staining or anti-goat polyclonal anti-snail (T-18), for cellular staining using secondary antibody conjugated to Alexa Fluor 488 (green) and analyzed by flow cytometry.

In summary, current colon cancer chemopreventive agents have a number of limitations. PEG appears to have significant efficacy with a desirable safety profile. Our current understanding of PEG’s cellular and molecular mechanisms is likewise promising. Our studies, therefore, provide an important direction for mechanistically relevant secondary endpoints.

Ability to Detect Putative Biomarkers in the Uninvolved Human Mucosa

Given that we are anticipating that three of the immunohistochemical markers will decrease with treatment, we assessed expression of Ki-67, SNAIL and EGFR in the normal mucosa of patients harboring neoplasia (similar to the study design protocol). In our preliminary studies we have noted there was clear, specific staining in the epithelium (data not shown).

Normal appearing mucosal biopsies may also have genetic signatures for CRC development. A/J colons elevated levels of genes such as Pole2, Hes6 and sf3b1 were observed in hyperplastic and dysplastic ACF, microadenomas, and adjacent normal appearing epithelium.(58)

Impact on Chemoprevention: The PEG doses used in this trial are associated with an excellent toxicity profile. Further, preclinical data supports the notion of significant chemopreventive activity. The current study offers an avenue to prospectively evaluate PEG chemopreventive efficacy in humans. If successful, these investigations will ultimately provide an effective and tolerable chemopreventive agent for a major cause of cancer-associated morbidity and mortality. Associated secondary endpoints are designed to evaluate relevant associated mechanisms, thereby elucidating both underlying cancer biology and molecular pharmacology.

- - 1. Rationale for Proposed Biomarkers

As discussed in our previous protocol, there are no unequivocally established intermediate biomarkers for colon carcinogenesis chemoprevention protocols. We, therefore, propose to use a panel of three markers that are accepted in the chemoprevention community (ACF, proliferation and apoptosis) and two markers that may be more specific to PEG (EGFR and SNAIL).

Epidermal growth factor receptor expression (EGFR)

There is unequivocal evidence of the importance of EGFR in established colon cancers. It is estimated that 70-80% of colon cancers overexpress EGFR. (76) In mice models, EGFR activation is critical for the earliest stages of carcinogenesis (microadenoma formation). (77) However, emerging evidence supports the role of EGFR in early colon cancer. For instance, Cohen and colleagues noted a marked overexpression of EGFR in human ACF.(78) Even in the histologically normal mucosa, rectal EGFR expression has been shown to correlate with proximal neoplasia.(79) The importance of EGFR is suggested by reports indicating that anti-EGFR strategies were able to suppress formation of 96% of intestinal adenomas. (57) While EGFR has not been well-studied as a chemopreventive biomarker, reports indicate that agents such as NSAIDs may prevent neoplasia at least partly through EGFR inhibition.(80) Our experimental data underscore the importance of EGFR in PEG-chemoprevention by demonstrating that not only is EGFR suppressed by PEG in the uninvolved mucosa but mitigating this suppression markedly decreased PEG efficacy. Thus, our use of EGFR in these studies is not simply as an intermediate biomarker but more to further study mechanism of action (e.g. dose levels of EGFR suppression correlate with other biomarker decrease). The literature and our preliminary data demonstrate that EGFR is detectable by immunohistochemistry in the uninvolved mucosa of humans.

Figure 7

Aberrant Crypt Foci (ACF) as a CRC Endpoint Biomarker

Since the initial identification of ACF as the premalignant morphological precursors of colon cancer 20 years ago, (59) ACF have been well established as a biomarker for colon carcinogenesis in both animal models and humans. The typical definition of ACF, as identified by high magnification chromoendoscopy are a heterogeneous group of abnormal crypts that are characterized by hyperproliferation, increased size, expanded peri-cryptal regions, and elongated or serrated lumens. Recent studies have show that there are gene expression alterations seen in ACF with most harboring K-ras mutations along with a myriad of other changes.(2, 60) Thus ACF are believed to be the earliest identifiable morphological lesions in colon carcinogenesis.

Studies have correlated the number of ACF (particularly in the rectum) with the presence of more advanced neoplasia (adenomas and carcinomas). In addition to several animal studies, relevance of ACF number and size has been tested in a number of epidemiological studies (61). Shpitz et al(62) also reported a higher prevalence of ACF in colon cancer patients and also found that ACF number was higher in distal than proximal colon. In another study, Bouzourene et al (63) using resected colons from CRC patients reported that the number of dysplastic ACF was higher in the CRC patients.

Use of magnification colonoscopy has demonstrated this in vivo. For instance, in a landmark article, Takayama and colleagues (46) noted that patients without neoplasia averaged ~1 ACF per rectum whereas those with adenomas or carcinomas were ~5 and ~25 rectal ACF respectively. Rudolph et al (64) recruited 32 patients who were scheduled for colonoscopy for a variety of indications such as history of polyps or GI bleed and found a significant elevation of ACF number in patients with personal history of adenoma. Seke et al (65) using data from 386 colonoscopy patients observed that ACF counted in the distal 15 cm of the rectum was a good predictor of advanced neoplasia throughout the colon/rectum.

There have been a plethora of studies using ACF as intermediate markers of chemoprevention in experimental models. Indeed, the correlation with adenomas and carcinomas appear excellent with a wide variety of agents (NSAIDs, PEG, etc).(66) Studies in humans have been limited. In the classic study by Takayama(46), 9 month therapy with sulindac markedly suppressed rectal ACF. In a more recent study, a significant decrease was noted within 2 months of therapy.(2) Shpitz et al(45) reported a 47% decline in the prevalence of ACF in colonic samples of patients treated with aspirin.

On the other hand, there have been some studies which have failed to show any association with rectal ACF and concurrent neoplasia. Adler and colleagues (67) noted that patients who were endoscopically normal had less ACF than those who harbored cancer (3.5 vs. 7.5, p<0.05) but were not different from patients who had adenomas (4.0). Moxon and colleagues (68) noted that smoking and age were determinants of rectal ACF levels. The limitations were well outlined in a recent review by Gupta and colleagues.(69)

Taken together, the biological relevance, prevalence studies and chemopreventive studies (both in experimental models and humans) strongly argue that ACF are the most robust marker for short term chemopreventive studies. While imperfect it would be an important endpoint prior to embarking on the gold standard studies – long term adenoma prevention studies. Even this endpoint is far from unassailable given the adenoma miss rate on colonoscopy and the fact that the vast majority of adenomas (or even advanced adenomas) will never develop into colon cancer.

Proliferation (Ki-67)

Diffuse mucosal hyperproliferation is one of the hallmarks of the field effect of colon carcinogenesis. Increased rectal proliferation has been shown to predict neoplasia throughout the colon (70). Indeed evaluation of proliferation has been a common means of gauging chemopreventive efficacy. From a teleological perspective, this mucosal hyperproliferation is critical for allowing expansion of initiated clones of cells. In experimental models, PEG has been shown to be associated with decreased mucosal proliferation. While a number of studies have raised concerns about the validity of exclusively relying on mucosa proliferation (71), the weight of the evidence suggests that proliferation is a reasonable intermediate biomarker. Importantly, there are a number of studies (including our preliminary data) that indicates that Ki-67 can be detected in the uninvolved mucosa (72, 73) thus providing the ability to detect a potential suppression with PEG.

Apoptosis (cleaved caspase-3)

The suppression of apoptosis is one of the critical early events in colon carcinogenesis allowing the otherwise short-lived colonocytes the requisite time to accumulate the mutational events to progress through the multistage neoplastic transformation process. The inhibition of apoptosis in the histologically normal rectosigmoid mucosa has been shown to predict the occurrence of neoplasia throughout the colon. (74). Cleaved caspase-3 is a well established means of gauging apoptosis in chemoprevention studies such as NSAIDs. (75) Of the means used to evaluate apoptosis, our group has found that cleaved caspase-3 is one of the most robust.(55) We have previously published that PEG induced apoptosis occurs in both cell culture and experimental models using cleaved caspase-3 along with other methods (TUNEL, etc.) (15, 49)

SNAIL

Our group and others have observed that the transcriptional repressor SNAIL is overexpressed in the majority of human colon cancers. (54) Importantly, we have published that in both the AOM-treated rat and the MIN mouse, SNAIL expression is detectable in the uninvolved mucosa (16, 55). Moreover, inhibiting SNAIL resulted in decreased MIN mouse tumors, supporting the role of SNAIL in initiation of tumorigenesis (55). As we have noted in the preliminary data, PEG treatment was associated with a marked inhibition of SNAIL levels. Our preliminary data indicates that the SNAIL immunoreactivity is noted in the uninvolved human colonic mucosa. Thus, our use of SNAIL in the studies is not simply as another (albeit unvalidated) intermediate biomarker of colon carcinogenesis but rather as a more specific gauge of the signaling cascades targeted by PEG.

1. **SUMMARY OF STUDY PLAN**

3.1 Study Design:

This is a randomized, double-blind, placebo-controlled phase 2 trial. Patients undergoing colonoscopy will have rectal ACF counted. Patients, regardless of ACF count, will be randomized in a 1:1:1 fashion to placebo (maltodextrose powder), to 8g/day PEG 3350 (sub-laxative dose), or to 17g/day PEG 3350 (laxative). Patients will undergo biopsy X 6 of normal mucosa (i.e., non-ACF sites) and up to 2 biopsies of mid-sigmoid ACFs (if present). After 6 months on treatment, patients will undergo flexible sigmoidoscopy with repeat rectal ACF count and 6 rectal biopsies of normal mucosa, up to 2 rectal ACF biopsies (if present) and up to 2 mid-sigmoid biopsies of ACF (if present) for analysis of secondary endpoints.

- 1. Number of Participants:

Sixty participants will complete the entire protocol. Approximately 140 subjects undergoing colonoscopy for a history of neoplasia will be screened. 105 subjects meeting inclusion and exclusion criteria will be randomized to one of three treatment arms – 17g PEG, 8g PEG or placebo. 10 participants in each treatment arm are anticipated to drop out over the 6 months of treatment, leaving 20 evaluable patients per arm. We expect 35 screening failures. Assuming a screening rate of approximately 2 patients per week, 140 patients will be consented in 70 weeks (approximately 18 months).

- 1. Brief Description of Study Population:

The study population includes men and women of all races and ethnicities who are scheduled to undergo colonoscopy for a history of colonic neoplasia (within the past 6 years of either colonic adenoma ≥ 5 mm or carcinoma).

3.4. Intervention plan, including doses, dose groups, and duration of exposure to the study agent:

Participants will take study agent for 6 months +/- 2 weeks. Participants will be sent a 200-day supply of study agent as single-dose powder sachets when randomized. Based on the patient’s randomization assignment, they will be provided with 17g of PEG, 8g of PEG or placebo powder sachets.

At baseline, after documentation of written informed consent, a medical history, physical exam, inclusion/exclusion criteria assessment, and clinical lab studies (CBC with differential, Chem Panel, INR and pregnancy test, if applicable) will be performed. The patient will undergo a colonoscopy, ACFs will be counted and biopsies will be taken. Eligible patients will be randomized and, within 6-10 days after colonoscopy, provided with study drug. Patients will be contacted by telephone at weeks 1, 2 and 4 to assess safety and compliance for the first month and on a monthly basis thereafter. Patients will return to the clinic when they have been on drug for 6 months for a flexible sigmoidoscopy, ACF count, biopsies, clinical labs and safety assessment. Patients will be contacted again 30 days after their last dose of study drug for safety assessment.

3.5. Description of Run-in period, if applicable:

There will be no run-in period. Patients will not begin taking study drug until at least the sixth day after their colonoscopy to allow the patient’s bowel habits to return to normal and for any procedure-related discomfort to subside.

- 1. Time points for performing study assessments

Study assessments will be performed at time of initial consent colonoscopy and 6 months later during flexible sigmoidoscopy. Research blood and biopsies will be taken at that time. In order to assess safety and compliance, patients will be contacted by telephone at weeks 1, 2, 4, 8, 12, 16 and 20.

3.7 Description of measurements taken to meet study objectives

- ACF Analysis: Patients undergoing colonoscopy will have rectal ACF analyzed by chromoendoscopy. After 6 months on treatment, patients will undergo flexible sigmoidoscopy with repeat rectal ACF determination.
- Biopsies: Patients will undergo biopsy X 6 of normal mucosa (i.e., non-ACF sites) and up to 2 sigmoid ACFs, if present. After 6 months on treatment, patients will undergo flexible sigmoidoscopy and 5 rectal biopsies of normal mucosa will be taken for secondary endpoint analysis. In addition, up to 2 rectal ACF and up to 2 sigmoid ACF biopsies will be performed. At each time point, 2 of the 6 rectal biopsies will be paraffin-embedded for assessment of Ki-67 and cleaved caspase-3 markers of proliferation and apoptosis, respectively and also for analysis of EGFR, SNAIL and E-cadherin expressions. The 3 other rectal biopsies will undergo protein and RNA extractions for Western blot and RT-PCR analyses.

ACF biopsies will be paraffin embedded and sliced into multiple sections for immunohistochemical staining of Ki-67, caspase-3, Snail, E-cadherin and EGFR. As the ACF sample may not be enough, we won’t be doing any protein measurements by Western blotting.

- 1. Description of clinical procedures**,** lab tests or other measurements taken to monitor effects of study agent on human safety and to minimize risks

PEG is widely used as a laxative and has been shown to have no absorption and hence is without potential systemic toxicity. Indeed, it is currently approved for over the counter. We will need to be cognizant of the possibility of PEG-induced diarrhea. Moreover, we need to mitigate any procedure related risk.

1. Diarrhea:

We believe the risk of diarrhea will be related to baseline characteristics of patients. Thus, we will use >2 bowel movements per day baseline as an exclusion criteria. We have selected two doses. The higher dose (17g) is the starting dose for the treatment of chronic constipation. As discussed in section 2.2, therapy for a month may result in some tachyphylaxis. However, we would anticipate that there may be some minor changes in bowel habits in many patients. We will also assess a dose that is ~50% of the initial clinical dose. We anticipate that this will not affect bowel habits based on work done with low dose PEG, specifically with healthy volunteers (as opposed to subjects with constipation). This has been reviewed by Chaussade .(18) Importantly, since they reported that “low dose PEG 4000 (<10g per day) did not modify stool weight or CTT” (Colonic transit time), we believe that the 8 g dose of PEG 3350 should not have a marked effect on bowel habits, and thus would be ideal for chemoprevention in the non-constipated patient. We will thus test whether this dose is sufficient for chemoprevention. It is important to note that in the pre-clinical models, sub-laxative doses of PEG appeared to offer anti-neoplastic benefits.

1. Coagulopathy

Since biopsies will be taken as part of the protocol, any patients with coagulopathy or on long term anti-coagulation (INR>1.5 or platelets< 100,000/µl) will be excluded. Since the American Society of Gastrointestinal Endoscopy position paper has indicated that there are no safety concerns performing cold biopsies in patients on nonsteroidal anti-inflammatory drugs or aspirin, this will not be an exclusion criterion.

c. Hypersensitivity

Participants with previous side-effects from PEG or methylene blue will be excluded from the study at initial consent. During the telephone contacts weeks 1, 2, 4, 8 and 16, patients will be questioned about any hypersensitivity symptoms.

d. Pregnancy

While pregnancy is not an absolute contraindication to PEG, it would be imprudent to perform elective endoscopy. Endoscopy is acceptable if clinically indicated on pregnant patients. Thus, we will perform a pregnancy test on women of child-bearing potential, defined as women who have not been surgically sterilized and have menstruated within the past year. We also will stipulate the need for birth control and ask about pregnancy during telephone contacts weeks 1, 2, 8, 12, 16 and 20 and during clinic visit week 4. Patients will be questioned about continued birth control usage as well as if they know/suspect that they might be pregnant. Based on these conversations, if there are any concerns about potential pregnancy, a serum pregnancy test will be obtained within one week of the telephone contact. Patients who become pregnant during the study will be withdrawn.

3.9 Duration of study

Based on the anticipated accrual rate of 2 patients per week, all patients are expected to be accrued within 18 months and all assessments are expected to be complete within 26 months of initiation.

1. **PARTICIPANT SELECTION**

**4.1** **Inclusion Criteria**

4.1.1 History of **any size** adenoma, known adenoma on present exam, or colon cancer within the last **6** years.

4.1.2 Scheduled for colonoscopy.

4.1.3 Ability to understand and the willingness to sign a written informed consent document.

4.1.4 Willingness to forego PEG laxative during the study period. If the patient has been on a consistent dose of non-PEG laxative for 90 days prior to study entry, the participant may continue those laxatives. Participants must agree to restrict additional laxative use to the rescue medication (bisacodyl) provided.

4.1.5 Age ≥ 18 years. Because no dosing or adverse event data are currently available on the use of Polyethylene Glycol 3350 in participants <18 years of age, children are excluded from this study but will be eligible for future pediatric trials, if applicable.

4.1.6 ECOG performance status 0-1 (equivalent to Karnofsky ≥70%; see Appendix A)

4.1.7 Participants must have normal organ and marrow function as defined below:

Leukocytes ≥3,000/μL

Absolute neutrophil count ≥1,500/μL

Platelets ≥100,000/μL

INR ≤ 1.5

Total bilirubin ≤1.5 X institutional ULN

AST (SGOT)/ALT (SGPT) ≤1.5 X institutional ULN

estimated glomerular filtration rate (eGFR). >45

BUN <40

4.1.8 The effects of PEG 3350 on the developing human fetus at the recommended therapeutic dose are unknown. For this reason, women of child-bearing potential must agree to use adequate contraception (hormonal or barrier method of birth control; restricting intercourse to a surgically sterilized partner; abstinence) for the duration of study participation. Should a woman become pregnant or suspect she is pregnant while participating in this study, she should inform her study physician immediately.

4.1.9 If patients are on a dose of cardioprotective aspirin, they must have been on a stable dose for three months prior to colonoscopy and agree to remain at that dose for the six months duration of the study. In addition, patients must agree to limit therapeutic NSAID use (e.g. pain relief) to no more than 30 cumulative days during the six month duration of the trial.

**4.2 Exclusion Criteria**

- - 1. Average of >2 bowel movements per day for the 90 days preceding study entry as assessed by self-report at baseline.
    2. Average consistency of stools described as watery or loose for the 90 days preceding study entry as assessed by self-report at baseline.
    3. Systemic chemotherapy for any cancer within 18 months prior to enrollment or evidence of active malignant disease.
    4. Radiation to the rectum within 24 months prior to enrollment.
    5. Polyethylene glycol use within 3 months of enrollment (except as part of colonoscopy preparation).
    6. Systemic corticosteroid use.
    7. Anticoagulant therapy.
    8. Inflammatory Bowel Disease.
    9. Removal of the rectum
    10. Evidence of proctitis (radiation, IBD, infectious, etc.) by history or endoscopy.
    11. Other investigational agent use within 30 days prior to enrollment.
    12. History of adverse reactions attributed to compounds of similar chemical or biologic composition to polyethylene glycol, bisacodyl or methylene blue.
    13. Uncontrolled intercurrent illness including, but not limited to, ongoing or active infection, symptomatic congestive heart failure, unstable angina pectoris, cardiac arrhythmia, or psychiatric illness/social situations that would limit compliance with study requirements.
    14. Pregnancy.

Pregnant women are excluded from this study because elective flexible sigmoidoscopy is contraindicated for pregnant women.

4.2.15 Patient must not have used suppository medication or enemas for the three months prior to the trial or for the duration of the trial except as directed for colonoscopy or flexible sigmoidoscopy procedure bowel preparation.

**4.3 Inclusion of Women and Minorities**

Both men and women and members of all races and ethnic groups are eligible for this trial.

**4.4 Recruitment and Retention Plan**

NorthShore University HealthSystem (NUH), University of Chicago, and Boston University are major teaching hospitals. All sites perform a large volume of endoscopy procedures (10,000 and 13,000 per year respectively) with the vast majority of procedures being colonoscopies. NUH has pioneered full integration of the electronic medical record (EPIC), which allows ready access to endoscopists’ schedules and patient medical records. Thus potentially eligible subjects can be identified and contacted prior to the procedure. Each of the three recruitment sites will have a 50% clinical coordinator who will work to identify and recruit eligible patients. The University of Chicago site is of particular importance in ensuring adequate minority participation due to its patient population.

Northwestern University will work closely with NUH, University of Chicago, and Boston University on an ongoing basis to monitor recruitment and adjust or employ new strategies as needed to maximize patient recruitment with an emphasis on ensuring the representation of women and racial and ethnic minorities. This assistance will include coordinating recruitment material design and placement.

Participant retention will be maximized by frequent patient contact during the early portion of the trial. During each telephone contact patients will be assessed for adverse events and counseled on AE management and the use of rescue medication.

**5. AGENT ADMINISTRATION**

Intervention will be administered on an outpatient basis. Reported adverse events and potential risks are described in Section 6.2.

**5.1 Dose Regimen and Dose Groups**

Group A: PEG 3350 17g dose once daily for 6 months.

Group B: PEG 3350 8g dose once daily for 6 months

Group C: Matching placebo (maltodextrin) 8g dose once daily for 6 months.

- 1. **PEG 3350 Administration**

Patients will self-administer PEG 3350 or placebo on an outpatient basis. Patients will dissolve one single-dose sachet of the provided powdered study agent in 8 oz. of water, juice, soda, coffee or tea once daily. Patients will be instructed to take the study agent at approximately the same time each day, 2 hours prior to eating.

Participants will be shipped a 200-day supply of study agent upon randomization. The study agent will be provided as single-dose sachets. These sachets will be provided by NCI DCP.

- 1. **Run-in Procedures**

There will be no run-in for this protocol.

- 1. **Contraindications**

Contraindications to PEG use are limited to a history of baseline diarrhea, known or suspected bowel obstruction or allergy to PEG.

Contraindications to the research ACF count and elective endoscopy procedures include methylene blue allergy, coagulation disorders or anticoagulant use, concomitant systemic corticosteroid use and pregnancy.

- 1. **Concomitant Medications**

Patients must not take any exclusionary medications such as anticoagulants or corticosteroids for the duration of the study.

Patients on a stable dose (consistent for 90 days prior to study entry) of non-PEG laxatives at the beginning of the study will be allowed to continue those medications. Rescue medication for constipation will be provided for all patients after the completion of the colonoscopy. If patients experience severe discomfort or have not had a bowel movement in 4 days, they will be instructed to take 10mg (2-5mg tablets) of bisacodyl. Patients are not to take any PEG-containing laxatives for the duration of the study. Patients will be given an instruction sheet to assist them in making decisions regarding laxative use.

Therapeutic NSAID use will be allowed but must not be used for a cumulative total of more than 30 days during the treatment. Cardioprotective aspirin use will be permitted as long as the patient has been on a stable dose for at least 3 months prior to entering the trial and remains on that stable dose throughout the trial. In addition occasional use of NSAIDS should be discouraged unless necessary (e.g. occasional headaches, menstrual cramps, muscle or joint pain, etc....) and well documented through out the study by the patient and the study staff and must not be used for a cumulative total of more than 30 days during the treatment.

All medications (prescription and over-the-counter), vitamin and mineral supplements, and/or herbs taken by the participant within 30 days prior to study entry through study the 30 day follow-up contact will be documented on the concomitant medication CRF and will include: 1) start and stop date, dose, units, frequency and indication. Medications taken for a procedure (e.g., biopsy) will also be included.

**5.6 Dose Modification**

All adverse events will be graded according to the NCI Common Toxicity Criteria, Version 3.0. All Grade 2 or higher toxicities judged by the investigator to be treatment-related will be considered dose-limiting toxicities (DLTs).

For subjects experiencing Grade 2 treatment-related toxicity, their treatment regimen will be adjusted to one dose every other day.

For subjects experiencing Grade 3 treatment-related toxicity, their treatment will be held. Once toxicity falls below grade 2, their regimen will be adjusted to one dose every other day. If the patient continues to experience Grade 2 toxicity for two weeks on every other day dosing, they will be instructed to discontinue study agent.

Subjects experiencing grade 2 or greater toxicity on every other day dosing, will be instructed to discontinue drug.

Subjects who experience toxicity above grade 3 will be instructed to discontinue drug.

Subjects who miss >4 weeks of therapy due to toxicity will be instructed to discontinue drug.

Any patient experiencing diarrheal symptoms on the Victoria Bowel Performance Scale (BPS) of +2 or greater will be counseled regarding the possibility that any increase in stool volume or frequency is temporary and encouraged to remain at the assigned dose level. If the patient is on a stable dose of laxatives and experiences diarrhea during treatment, the patient will be instructed to discontinue the laxatives. If the patient continues to find the increase unacceptable after 1 week, their regimen will be adjusted to one dose every other day.

**5.7 Adherence/Compliance**

5.7.1 Patients will be considered compliant if they take at least 80% of their prescribed doses.

5.7.2 Compliance will be assessed using patient diary information.

**6. PHARMACEUTICAL INFORMATION**

**6.1 Polyethylene Glycol 3350**

Polyethylene Glycol 3350 (generic formulation of Miralax) will be used in this study. This formulation is FDA-approved and widely used in the United States for the treatment of constipation.

Polyethylene Glycol 3350 is a synthetic polyglycol having an average molecular weight of 3350. Actual molecular weight is not less than 90.0% and not greater than 110.0% of the nominal value. The chemical formula is HO (C_2_H_4_O)_n_H in which n represents the average number of oxyethylene groups. Below 55°C it is a freely flowing white powder freely soluble in water.

6.2 Reported Adverse Events and Potential Risks

Polyethylene glycol 3350 (17g/day) is a widely used prescription agent for the treatment of occasional constipation. Nausea, vomiting, abdominal bloating, cramping and flatulence may occur. Patients taking other medications containing PEG have occasionally developed urticaria suggestive of an allergic reaction. Very rarely described adverse events include Mallory-Weiss tear, lung edema, increased ventricular arrhythmia, and anaphylactic reaction.

While PEG 3350’s side effect profile in constipated patients is well-characterized, less is known about the use of PEG 3350 in non-constipated patients. The risk of diarrhea is expected to be minimal and will be more of a nuisance symptom than a significant health concern. To minimize the incidence of diarrhea, patients with >2 bowel movements per day at baseline will be excluded. All patients will be monitored for change in bowel habits and personal assessments of GI discomfort. The dose modification plan is described in section 5.6.

The starting dose of PEG 3350 for patients with clinical constipation is 17g and would be anticipated to cause minor changes in bowel habits in many patients. In a multicenter, placebo-controlled trial with 304 patients meeting Rome 2 criteria, the weekly bowel movement total was greater for PEG than placebo after 6 months of therapy (7.9 versus 5.6, p< 0.001). It is important to note that this increase is less than 0.5 bowel movements per day. There were no treatment emergent safety differences between PEG and placebo over the course of the 6-month study except for gastrointestinal complaints (PEG 39.7%, placebo 35%, P = 0.015). Although not individually statistically significant, the difference was driven by abdominal distension, diarrhea, loose stools, flatulence, and nausea. These effects are consistent with and expected from laxative therapy. Most of these events were mild or moderate. Two cases of diarrhea were rated “severe”, one resolved spontaneously, the other upon PEG discontinuation.(17) In another recent study by the same authors of 311 patients meeting Rome 2 criteria for constipation (~1/3 considered elderly), they found that PEG efficacy and tolerability seemed to be stable over a 12 month time span. (43)

While PEG 3350 is indicated for short term treatment of constipation, the data on long term safety has been supported by recent publications.(43) In an open label, single treatment multi-center study, 311 patients received 17 g of PEG 3350 for 12 months. In this study, 184 patients completed until Day 360. Of the dropouts, 23 dropped out for adverse events. The majority of these occurred within the first period with much less attrition at longer time points. In the population, 2.9% had abdominal distention, 10.6% had diarrhea, 7.4% had flatulence, 3.9% had loose stools and 5.5% had nausea. Importantly, no serious adverse event was considered related to study medication. Of the patients who dropped out, the majority (21 out of 23) withdrew because of diarrhea, distention, nausea and flatulence. Based on the data presented in the manuscript, there were no SAEs or laboratory changes observed.

The 8g dose is approximately 50% of the initial clinical dose and will also be assessed. Bowel habits are not anticipated to be affected by the 8g dose based on work done with low dose PEG 4000 on healthy, non-constipated, volunteers. Specifically, PEG 4000 doses of less than 10g did not modify stool weight or colonic transit time.(43) As the 8g dose shows chemopreventive efficacy in pre-clinical models, but does not appear to have a marked effect on bowel habits in non-constipated patients, this may be the ideal dose for chemoprevention in healthy prevention patients.

**6.3 Availability**

PEG 3350 and placebo (maltodextrin) will be supplied by NCI, DCP.

**6.4 Agent Distribution**

NCI, DCP will purchase and package the drug and placebo.

**6.5 Agent Accountability**

The Investigator, or a responsible party designated by the Investigator, must maintain a careful record of the inventory and disposition of all agents received from DCP using the NCI Drug Accountability Record Form (DARF). The Investigator is required to maintain adequate records of receipt, dispensing and final disposition of study agent. Include on receipt record from whom the agent was received and to whom study agent was shipped, date, quantity and batch or lot number. On dispensing record, note quantities and dates study agent was dispensed to and returned by each participant.

- 1. **Packaging and Labels**

PEG 3350 and matching placebo will be packaged by NCI, DCP. NCI, DCP will be responsible for distributing the study agents to University of Chicago, Boston University and NorthShore University HealthSystem sites.

Each patient’s drug supply will be provided as a 200-day supply of single-dose powder sachets. Each sachet will be labeled with a one-part label identifying study specific information, such as study title, DCP protocol number, dosing instructions, recommended storage conditions, the name and address of the distributor, randomization number, and a caution statement indicating that the agent is limited by United States law to investigational use only and the agent should be kept out of reach of children. To maintain the study blind, the study agent will be described as “Polyethylene Glycol 3350 or placebo”.

- 1. **Storage**

Both PEG 3350 and placebo (maltodextrin) should be stored at room temperature – between 59°F and 86°F under low humidity.

**6.8 Registration/Randomization**

All patients will be registered to the study through Northwestern University as the central coordinating site. All subjects are to be registered with the CLO Site Coordinator. The completed informed consent and patient registration form are to be faxed to (312) 695-1352.

Patients who have met all study entry criteria will be randomized by Northwestern University’s Clinical Research Office. Someone will call or page the Northwestern University QA Coordinator to randomize a patient at (312) 695-1355 between 8:30am and 5:30 pm. Once study eligibility has been confirmed, the QA coordinator will randomize the patient and provide the randomization ID number to the study coordinator. Investigators and subjects will be blinded as to the result of randomization. To begin the study protocol of the investigational agent, the patients will be provided with study agent 6-10 days post initial colonoscopy to ensure that all normal post endoscopy discomforts are resolved and that the patient does not ascribe those discomforts to the drug.

6.9 Blinding and Unblinding Methods

The Principal Investigator and research coordinator will manage the investigational agent. Study participants will receive a prescription, in a blinded fashion, from the Principal Investigator. The blind will be maintained through the effort of Northwestern’s Quality Assurance Manager. Unblinding will only occur when it is deemed medically necessary, and will only take place after consultation with the NCI, DCP Medical Monitor Luz Rodriguez, MD (Emergency after-hours contact: 301-693-2620). If the NCI medical monitor cannot be reached and the participant requires emergency care, the Study Chairman may authorize the site PI to break the blind. The date and reason for breaking the blind must be submitted by the site PI to the Study Chairman and the NCI Medical Monitor as soon as possible.

**6.10 Agent Destruction/Disposal**

At the completion of the investigation, all returned or unused study agent will be disposed of according to institution-specific policy and procedures for handling investigational agents. Each institution will maintain a record of what is destroyed including name of agent, quantity, lot number and expiration date. All records will be maintained for 5 years from when the study is completed.

**7. CLINICAL EVALUATIONS AND PROCEDURES**

**7.1 Schedule of Events**

| **Evaluations / Procedures** | **Pre-Study** | **Randomization** | **Day 0** | **1 Week** | **2 Weeks** | **1 Month** | **2 Months** | **3 Months** | **4 Months** | **5 Months** | **6 Months / Early Termination** | **30 day post-treatment follow-up** |
| --- | --- | --- | --- | --- | --- | --- | --- | --- | --- | --- | --- | --- |
| **Informed Consent** | **X** |  |  |  |  |  |  |  |  |  |  |  |
| **Medical History** | **X** |  |  |  |  |  |  |  |  |  |  |  |
| **Baseline Symptom Review** | **X** |  |  |  |  |  |  |  |  |  |  |  |
| **Physical Exam** | **X** |  |  |  |  |  |  |  |  |  | **X** |  |
| **Vital Signs assessment** | **X** |  |  |  |  |  |  |  |  |  | **X** |  |
| **Concomitant Medication Review** | **X** |  | **X** | **X** | **X** | **X** | **X** | **X** | **X** | **X** | **X** | **X** |
| **Inclusion/Exclusion Criteria Review** | **X** | **X** |  |  |  |  |  |  |  |  |  |  |
| **Clinical Lab Tests** | **X** |  |  |  |  |  |  |  |  |  | **X** |  |
| **Research Blood** | **X** |  |  |  |  |  |  |  |  |  | **X** |  |
| **Colonoscopy** | **X** |  |  |  |  |  |  |  |  |  |  |  |
| **Rectal Biopsies** | **X** |  |  |  |  |  |  |  |  |  | **X** |  |
| **Flexible Sigmoidoscopy** |  |  |  |  |  |  |  |  |  |  | **X** |  |
| **Registration** | **X** |  |  |  |  |  |  |  |  |  |  |  |
| **Randomization** |  | **X** |  |  |  |  |  |  |  |  |  |  |
| **Rescue Medication (Bisacodyl) Dispensing** | **X** |  |  |  |  |  |  |  |  |  |  |  |
| **Study Drug Shipment** |  | **X** |  |  |  |  |  |  |  |  |  |  |
| **Study Drug Start** |  |  | **X** |  |  |  |  |  |  |  |  |  |
| **Telephone Contact** |  |  | **X** | **X** | **X** | **X** | **X** | **X** | **X** | **X** |  | **X** |
| **Diary Distribution** | **X** |  |  |  |  |  |  |  |  |  |  |  |
| **Diary Review** |  |  | **X** | **X** | **X** | **X** | **X** | **X** | **X** | **X** | **X** | **X** |
| **Adverse Events Assessment** |  |  | **X** | **X** | **X** | **X** | **X** | **X** | **X** | **X** | **X** | **X** |
| **Collect and Count Study Agent** |  |  |  |  |  |  |  |  |  |  | **X** |  |

**7.2 Baseline Testing/Pre-study Evaluation**

Pre-Study Evaluations

- Informed Consent – written informed consent must be obtained prior to any other study procedure
- Medical History and Baseline Symptom Review
- Concomitant Medication Review – all medications that the participant has taken within the 30 days prior to consent are to be recorded
- Physical Exam and Vital Signs assessment – vital signs assessment is to include pulse, blood pressure, respiration rate, temperature and weight. Baseline assessment must also include height.
- Inclusion / Exclusion Criteria Review
- Clinical Lab Tests – CBC with differential and Chemistry Panel (White blood count, RBC count, Hemoglobin, Hct, Platelets, absolute neutrophil count, Glucose, Creatinine, BUN, eGFR, Sodium, Potassium, Chloride, SGOT, SGPT, Total Bilirubin). Baseline assessment must include an INR for all patients and a serum pregnancy test for all women of childbearing potential. Women of childbearing potential include those who have not been surgically sterilized and have had a period in the past year.
- Research Blood – Research blood will be collected: Peripheral blood mononuclear cells for RNA isolation and storage and plasma for storage.
- Colonoscopy with 6 rectal biopsies of non-ACF tissue.
- Dispense 30 5mg bisacodyl tablets for use as rescue medication. Bisacodyl should only be dispensed to patients with sufficient ACF who are expected to be randomized pending lab results.
- Register the patient with Northwestern University Clinical Research Office.

Randomization

- Confirm that the patient meets all inclusion/exclusion criteria
- Randomize the patient with Northwestern University Clinical Research Office.
- Ship initial supply of study agent (refer to section 5.2) and patient diary to participant.

**7.3 Evaluations During Study Intervention**

Day 0

- Telephone contact with participant with instructions to begin study agent and diary completion.
- Assess Adverse Events
- Assess Concomitant Medications

1 Week (+/- 3 days)

- Telephone contact with participant
- Assess study drug compliance
- Assess adverse events and discuss patient diary
- Assess Concomitant Medications

2 Weeks (+/- 3 days)

- Telephone contact with participant
- Assess study drug compliance
- Assess adverse events and discuss patient diary
- Assess Concomitant Medications

1 Month (+/- 3 days)

- Telephone contact with participant
- Assess study drug compliance
- Assess adverse events and discuss patient diary
- Assess Concomitant Medications

2 Months (+/- 7 days)

- Telephone contact with participant
- Assess study drug compliance
- Assess adverse events and discuss patient diary
- Assess Concomitant Medications

3 Months (+/- 7 days)

- Telephone contact with participant
- Assess study drug compliance
- Assess adverse events and discuss patient diary
- Assess Concomitant Medications

4 Months (+/- 7 days)

- Telephone contact with participant
- Assess study drug compliance
- Assess adverse events and discuss patient diary
- Assess Concomitant Medications

5 Months (+/- 7 days)

- Telephone contact with participant
- Assess study drug compliance
- Assess adverse events and discuss patient diary
- Assess Concomitant Medications
  1. **Evaluations at Completion of Study Intervention**

6 Months (+/- 14 days)

- Concomitant Medication Review
- Physical Exam and Vital Signs assessment – vital signs assessment is to include pulse, blood pressure, respiration rate, temperature and weight.
- Clinical Lab Tests – CBC with differential and Chemistry Panel (White blood count, RBC count, Hemoglobin, Hct, Platelets, absolute neutrophil count, Glucose, Creatinine, BUN, eGFR, Sodium, Potassium, chloride, SGOT, SGPT, Total Bilirubin).
- Research blood will be collected for PBMC analysis.
- Flexible sigmoidoscopy with 6 rectal biopsies of non-ACF tissue.
- Assess adverse events and review patient diary
- Collect and count study medication from patient
  1. **Post-intervention Follow-up Period**

Participants will be assessed 30 days after their last dose of the study agent for assessment of adverse events.

30 days post-treatment (+7 days)

- Telephone contact with participant
- Assess adverse events and discuss patient diary
- Assess Concomitant Medications
  1. **Methods for Clinical Procedures**

**Identification and Quantification of ACF *in situ***: ACF will be identified in the rectal mucosa by image magnification chromoendoscopy.(65) Endoscopists will be trained in the identification of ACF prior to the study. A video provided by the Mayo Clinic demonstrating the methylene blue dye spray protocol and real time identification of ACF will be viewed by all endoscopists involved in this study. Dr. Paul Limberg, who has extensive experience in this area, has discussed his protocol for ACF training and testing with us and he has agreed to provide any assistance we may need with this process. With the goal of reducing interobserver variability, one dozen photographs of rectal mucosa with varying numbers of ACF will be obtained using the same dye spray protocol and equipment described above. These photographs will be reviewed by each endoscopist who will be tested on the endoscopic findings. Accurate endoscopic appraisal of 10 of the 12 photos will be required of each endoscopist prior to participating in the study. Investigators who fail to meet this standard will be retrained and retested using different photos.

All patients will have a standardized preparation for the colonoscopy consisting of Trilytely (bisacodyl + 2-3 L polyethylene glycol) until clear.

After completion of colonoscopy, the endoscope will be advanced to the mid-sigmoid colon (approximately 35cm) and the sigmoid and rectal mucosa will be washed with tap water. The rectum will be defined as the segment from the dentate line to the rectosigmoid junction which is located approximately 13-15 cm from the anal verge. In cases where the rectosigmoid junction is not easily identified, the rectum will be defined as the segment from the dentate line to 15 cm from the anal verge. An Olympus PW-5V-1 spray catheter will be used to distribute 120 cc of mucomyst onto the sigmoid and rectal mucosa in an effort to dissolve any adherent mucous. This will be followed by washing the mucosa a second time with tap water. Finally, between 20-120 ml of freshly prepared 0.2% methylene blue solution will be dispersed over the sigmoid and rectal mucosa using the spray catheter. The volume of methylene blue may vary due to the goal of achieving an adequate dye spray. We will define an adequate dye spray as one in which > 90% of the rectal mucosa is penetrated with dye.

After removal of excess methylene blue via suction, the stained mucosa will be examined carefully while withdrawing the magnification colonoscope (Olympus 160 series), and the number of ACF counted. (78) Inspection of the rectal mucosa will begin at the proximal margin of the rectum in a counterclockwise manner and the entire rectal mucosa will be examined in 1 cm increments. ACF will be defined as a collection of crypts which are larger in diameter, stain more darkly, and have a thicker epithelium than normal crypts. The location from the anal verge of each ACF will be noted and photo documentation of each ACF will be obtained. The lead endoscopists will review these photos to confirm the presence of ACF. Based on existing data the inter-observer variability for determination of number of crypts within each ACF is high, therefore the number of crypts per ACF will be assessed in a semi-quantitative manner as follows: small ACF (0-10 crypts), medium ACF (11-20 crypts), large ACF (>20 crypts).

**Importantly, to decrease per-patient variability, when patients return for the 6 month evaluation, the same endoscopist who performs the pre-treatment ACF count will also perform the 6 month evaluation.**

**Research Flexible Sigmoidoscopy:**

Participants will be scheduled for research flexible sigmoidoscopy between 8 am and 10 am. The patients will fast after midnight. One Fleets enema will be administered 30 minutes prior to the flexible sigmoidoscopy to clear the bowel.

The participants will be placed on their left side and a rectal exam will be performed. The endoscope will be advanced to the mid-sigmoid colon (approximately 35cm) and the sigmoid and rectal mucosa will be washed with tap water. The rectum will be defined as the segment from the dentate line to the rectosigmoid junction which is located approximately 13-15 cm from the anal verge. In cases where the rectosigmoid junction is not easily identified, the rectum will be defined as the segment from the dentate line to 15 cm from the anal verge. An Olympus PW-5V-1 spray catheter will be used to distribute 120 cc of mucomyst onto the sigmoid and rectal mucosa in an effort to dissolve any adherent mucous. This will be followed by washing the mucosa a second time with tap water. Finally, between 20-120 ml of freshly prepared 0.2% methylene blue solution will be dispersed over the sigmoid and rectal mucosa using the spray catheter. The volume of methylene blue may vary due to the goal of achieving an adequate dye spray. We will define an adequate dye spray as one in which > 90% of the rectal mucosa is penetrated with dye.

After removal of excess methylene blue via suction, the stained mucosa will be examined carefully while withdrawing the magnification colonoscope (Olympus 160 series), and the number of ACF counted. (78) Inspection of the rectal mucosa will begin at the proximal margin of the rectum in a counterclockwise manner and the entire rectal mucosa will be examined in 1 cm increments. ACF will be defined as a collection of crypts which are larger in diameter, stain more darkly, and have a thicker epithelium than normal crypts. The location from the anal verge of each ACF will be noted and photo documentation of each ACF will be obtained. The lead endoscopists will review these photos to confirm the presence of ACF. Based on existing data the inter-observer variability for determination of number of crypts within each ACF is high, therefore the number of crypts per ACF will be assessed in a semi-quantitative manner as follows: small ACF (0-10 crypts), medium ACF (11-20 crypts), large ACF (>20 crypts).

Following ACF analysis 6 rectal biopsies will be taken of non-ACF containing areas (at least 1 cm from an ACF) and up to 2 mid-sigmoid and rectal ACFs, if available. Hemostasis will be confirmed, air removed and the procedure will be terminated (estimated time to complete procedure approximately 5 minutes).

**8. CRITERIA FOR EVALUATION AND ENDPOINT DEFINITION**

This study seeks to determine whether six months of daily PEG 3350 at 17g/day or 8g/day will decrease epidermal growth factor (EGFR) expression when compared to placebo. Comparisons will also be made between the 8g/day and 17g/day treatment arms. In addition, the effect of PEG 3350 on ACF count, SNAIL, and E-cadeherin will be assessed for support of the putative mechanism.

- 1. **Primary Endpoint**

The primary endpoint examined in this study is the difference A-B (After treatment minus Before treatment) of EGFR expression.  The difference in the observed change from baseline in each treatment arm will be compared with placebo.  EGFR will be measured by immunoblot. mRNA expression of EGFR will be measured by RT-PCR.

- 1. **Secondary Endpoints**

Secondary endpoints will include changes in ACF count, Ki-67 (proliferation), activated caspase-3 (apoptosis), SNAIL and E-cadherin as measured in endoscopically normal (non-ACF) mucosal biopsies. Ki-67, activated caspase-3, and SNAIL will be measured by IHC. EGFR and E-cadherin will be measured by immunoblot. mRNA expression of EGFR and SNAIL will be measured by RT-PCR.

- 1. **Off Agent Criteria**

Participants may stop taking study agent for the following reasons: the completion of the protocol-prescribed intervention, adverse event or serious adverse event, inadequate agent supply, noncompliance, concomitant medications, medical contraindication (including pregnancy), withdrawal of informed consent, or best medical interest of the patient as determined by the investigator, and/or the integrity of the data, to discontinue the study agent. Participants will continue to be followed, if possible, for safety reasons and in order to collect endpoint data according to the schedule of events. Subjects who stop treatment for toxicity reason will be followed until the issue is resolved or stabilized.

- 1. **Off Study Criteria**

Participants may go ‘off-study’ for the following reasons: the protocol intervention and any protocol-required follow-up period is completed, adverse event/serious adverse event, lost to follow-up, non-compliance, concomitant medication, medical contraindication (including pregnancy), withdrawn consent, or death. Participants may also go ‘off-study’ if the study is terminated.

- 1. **Study Termination**

NCI, DCP as the study sponsor has the right to discontinue the study at any time as does the FDA.

1. CORRELATIVE/SPECIAL STUDIES
   1. **Rationale for Methodology Selection**

Three of the six biopsies will be pooled and homogenized in 1 ml of Trizol Reagent to extract both RNA and protein from the same sample pool. RNA will then be subjected to RT-PCR to measure the SNAIL message since in our experience the commercial SNAIL antibody is not suitable for Western blotting.

EGFR and E-cadherin will be assessed by Western blot analysis for a more quantitative understanding. .

Three of the six rectal biopsies will be embedded for immunohistochemistry. IHC has the advantage of allowing identification of the cells being probed but has the disadvantage of being semi-quantitative with regards to intensity and expression. Colonic epithelial apoptosis and cell proliferation will be determined by cleaved caspase-3 and Ki-67, respectively. Caspase-3 and Ki-67 will be assessed using IHC since the percentage of positive cells is the gold standard for reporting these results. Cleaved caspase-3 and Ki-67 are robust, well-validated measures of these parameters. EGFR and SNAIL will also be assessed by IHC. For this we will use computer-aided quantitation to improve the robustness of expression analysis.

Peripheral blood mononuclear cell RNA, and blood plasma, will be stored and analyzed in the future for gene expression and proteomic analysis, respectively. The conduct of early phase cancer trials provides the opportunity to evaluate drug effect markers. Clinical samples will therefore be captured and stored for purposes of future molecular fingerprinting at the level of both the proteome as well as the level of gene expression (i.e. RNA). Therapy-induced changes in cellular function will be expected to result from changes in gene expression and/or protein expression. Advances in technology now permit protein and expressed gene (RNA) compartments to be analyzed on a broad scale, or to be fingerprinted. Pre- and post-treatment plasma and RNA from peripheral blood mononuclear cells will therefore be collected and stored for future batch analysis. Blood plasma proteins will be characterized by mass assisted laser desorption and ionization time of flight (MALDI-TOF) spectrometric analysis, while expressed genes will be characterized by using RNA to probe gene arrays. Samples will be banked, and processed in the future. Resultant data relating to the molecular effects of this drug, as well as future drugs, will be used to populate a library. This data library, which will not have any patient identifier information in it, will contain information which will cross classes of drugs, as well as target cohorts (i.e. cohorts at risk for different cancer types). There are currently no funds for completing the molecular profiling, or for creating the resultant database. Thus, samples will be collected and banked for this dedicated use.

- 1. **Comparable Methods**

Proposed methods represent standard technology for chemoprevention trials in the colon.

**10. SPECIMEN MANAGEMENT**

**10.1 Laboratories**

Clinical laboratory samples will be handled by the hospital laboratories at each site. Evanston Hospital, Glenbook Hospital, University of Chicago Hospital, and Boston University each maintain a certified clinical laboratory.

Northwestern University Pathology Core Facility (PCF) – The PCF is a large comprehensive core facility within the Robert H. Lurie Comprehensive Cancer Center of Northwestern University. The PCF is responsible for providing specimen collection kits, initial processing of many research specimens and blood and tissue banking. The PCF is located at 710 N. Fairbanks Ct., Olson 8501, Chicago, Illinois, 60611. The PCF manager can be contacted at telephone number (312) 908-9595 and pager number (312) 695-5802.

Dr. Bergan’s laboratory – This lab will be responsible for isolation of RNA and protein from peripheral blood mononuclear cells (PBMCs).

Drs. Roy and Wali’s laboratory – This lab will be responsible for IHC and immunoblot analyses as well as initial processing of PBMCs for freezing and later batch shipment to the PCF for storage and distribution to the Bergan laboratory. They will also be responsible for RT-PCR analyses. The laboratory is located at Boston Medical Center, 650 Albany Street, Boston, MA 02118. Dr. Wali can be reached at 617-638-8223.

**10.2 Collection and Handling Procedures**

30 ml of research blood for PBMC analysis and five rectal biopsies will be obtained at the pre-study and 6 month visit. Research blood for PBMC analysis will be processed at Evanston Hospital and Glenbrook Hospital to the point that the samples can be frozen for later batch shipment to the PCF. University of Chicago and Boston University will send the blood samples via courier to PCF for processing. The PCF will provide specimen collection kits with all sample collection, labeling and packaging information. Rectal biopsies will be placed in formalin overnight and sent to the PCF for processing and distribution.

The PCF has SOPs in place that conform to all federal, state, and local regulations. SOPs are updated regularly to ensure compliance with changing regulations. Personnel within the PCF receive initial and ongoing training to ensure competence and regulatory compliance. The PCF maintains a large number of secure remote alarm access storage devices. Individual samples are assigned a unique number, de-identified, and are tracked through a bar code mechanism.

- 1. **Shipping instructions**

The six rectal biopsies (from NorthShore, U of C, and Boston University) will be placed in formalin overnight and sent via courier to the PCF for paraffin embedding and slide preparation. After embedding, the samples will be sent to Dr. Wali’s laboratory by the PCF.

- 1. **Tissue Banking**

Tissue samples that are banked will be de-identified through established SOPs within the Pathology Core Facility (PCF) no information will be conveyed back to individual subjects, nor will any link to individual subjects be sought. Samples will be stored by the PCF.

Biologic specimens collected during the conduct of each clinical trial that are not used during the course of the study will be considered deliverables under the contract and thus the property of the NCI. At study completion, NCI reserves the option to either retain or relinquish ownership of the unused biologic specimens. If NCI retains ownership of specimens, the Contractor shall collect, verify and transfer the requested biologic specimens from the site to a NCI-specified repository or laboratory at NCI’s expense.

**11. REPORTING ADVERSE EVENTS**

DEFINITION: An adverse event (AE) is any untoward medical occurrence in a study participant. An AE does not necessarily have a causal relationship with the treatment or study participant. An AE can therefore be any unfavorable and unintended sign (including a clinically significant abnormal laboratory finding), symptom, or disease temporally associated with participation in a study, whether or not related to that participation. This includes all deaths that occur while a participant is on a study.

A list of adverse events that have occurred or might occur (Reported Adverse Events and Potential Risks) can be found in Section 6.2, Pharmaceutical Information as well as the Investigator Brochure or package insert.

**11.1 Adverse Events**

11.1.1 Reportable Adverse Events

All adverse events that occur after the informed consent is signed must be recorded on the adverse event CRF (paper and/or electronic) whether or not related to study agent. The only adverse events which will not be reported are normal post-endoscopy discomfort and bloating. Any discomfort, bloating or change in bowel habits from baseline present six days after colonoscopy will be reported as an AE. Adverse drug reactions will be reported to both the drug supplier and the NorthShore University HealthSystem IRB in writing within 10 working days of the event.

- - 1. AE Data Elements:
- AE reported date
- AE Verbatim Term
- CTCAE Term (v 3.0)
- Event onset date and event ended date
- Severity grade
- Attribution to study agent (relatedness)
- Whether or not the event was reported as a Serious Adverse Event (SAE)
- Action taken with the study agent
- Outcome of the event
- Whether the patient was dropped due to AE
- Comments

11.1.3 Severity of AEs

- - - 1. Identify the adverse event using the NCI Common Terminology Criteria for Adverse Events (CTCAE) version 3.0. The CTCAE provides descriptive terminology and a grading scale for each adverse event listed. A copy of the CTCAE can be found at [http://ctep.cancer.gov](http://ctep.cancer.gov/).

AEs will be assessed according to the CTCAE grade associated with the AE term. AEs that do not have a corresponding CTCAE term will be assessed according to their impact on the participant’s ability to perform daily activities as follows:

| Grade | Severity | Description |
| --- | --- | --- |
| 1 | Mild | - Barely noticeable, does not influence functioning - Causing no limitations of usual activities |
| 2 | Moderate | - Makes participant uncomfortable, influences functioning - Causing some limitations of usual activities |
| 3 | Severe | - Severe discomfort, treatment needed - Severe and undesirable, causing inability to carry out usual activities |
| 4 | Life threatening | - Immediate risk of death - Life threatening or disabling |
| 5 | Fatal | - Causes death of the participant |

11.1.4 Assessment of relationship of AE to treatment

The possibility that the adverse event is related to study drug will be classified as one of the following: not related, unlikely, possible, probable, definite.

11.1.5 Follow-up of AEs

All AEs, including lab abnormalities that in the opinion of the investigator are clinically significant, will be followed according to good medical practices and documented as such.

**11.2 Serious Adverse Events**

11.2.1 DEFINITION: Fed. Reg. 75, Sept. 29, 2010 defines SAEs as those events, occurring at any dose, which meet any of the following criteria:

• Results in death

• Is life threatening (Note: the term life-threatening refers to an event in which the patient was at risk of death at the time of the event; it does not refer to an event which hypothetically might have caused death if it were more severe).

• Requires inpatient hospitalization or prolongation of existing hospitalization

• Results in persistent or significant incapacity or substantial disruption of the ability to conduct normal life functions

• Is a congenital abnormality/birth defect

• Important medical events that may not result in death, be life-threatening or require hospitalization may be considered serious when, based upon appropriate medical judgment, they may jeopardize the patient or subject and may require medical or surgical intervention to prevent one of the outcomes listed.

11.2.2 Reporting Serious Adverse Events to DCP

- - - 1. The Lead Organization and all Participating Organizations will report SAEs to DCP according to the DCP Serious Adverse Event Reporting Procedures and Guidelines as posted in the Clinical Trials Resource on the DCP website.

11.2.2.2 Contact the DCP Medical Monitor by phone within 24 hours of knowledge of the event.

Luz Maria Rodriguez, MD, FACS

National Institutes of Health, National Cancer Institute

Division of Cancer Prevention

9609 Medical Center Drive, Room 5E228

Bethesda, MD 20892

Phone: 240‐276‐7039

Fax (with cover sheet, Attn: Dr. L. Rodriguez): 240‐276‐7848

Email (preferred): rodrigul@mail.nih.gov

Include the following information when calling the Medical Monitor:

- - - - Date and time of the SAE
      - Date and time of the SAE report
      - Name of reporter
      - Call back phone number
      - Affiliation/Institution conducting the study
      - DCP protocol number
      - Title of protocol
      - Description of the SAE, including attribution to drug and expectedness
      1. The Lead Organization and all Participating Organizations will FAX written SAE reports to the DCP Medical Monitor within 48 hours of learning of the event using the paper SAE form. The written SAE reports will also be FAX’ed to NCI’s Regulatory Contractor, CCS Associates, at (650)691-4410 (phone: (650)691-4400). They may also be emailed to the regulatory contractor at safety@ccsainc.com.

- - - 1. The DCP Medical Monitor and regulatory staff will determine which SAEs require FDA submission.
      2. The Lead Organization and all Participating Organizations will comply with applicable regulatory requirements related to reporting SAEs to the IRB/IEC.

11.2.2.6 Follow-up of SAE

Site staff should send follow-up reports as requested when additional information is available. Additional information should be entered on the DCP SAE form in the appropriate format. Follow-up information should be sent to DCP as soon as possible according to the DCP Serious Adverse Event Reporting Procedures and Guidelines. SAEs will be followed until they have resolved or have stabilized.

**12. STUDY MONITORING**

**12.1 Data Management**

This study will report clinical data using the DCP Oracle Clinical Remote Data Capture (OC-RDC) web-based application managed by DCP’s monitoring contractor. The OC-RDC will be the database of record for the protocol and subject to NCI and FDA audit. All OC-RDC users will be trained to use the RDC system and will comply with the instructions in the protocol-specific “RDC User Manual” provided to the Consortium Lead PI by the DCP Monitoring Contractor as well as applicable regulatory requirements such as 21 CFR; Part 11.

An approved Master Data Management Plan that is applicable to all studies within the Consortium will be on file at DCP.

**12.2 Case Report Forms**

Participant data will be collected using protocol-specific case report forms (CRF) developed from the standard set of DCP Chemoprevention CRF Templates and utilizing NCI-approved Common Data Elements (CDEs). The approved CRFs will be used to create the electronic CRF (e-CRF) screens in the OC-RDC application. Site staff will enter data into the e-CRF for transmission to DCP according to pre-established DCP standards and procedures. Amended CRFs will be submitted to the DCP Protocol Information Office for review and approval. Approved changes will be programmed into the OC-RDC database by the DCP Monitoring Contractor.

**12.3 Source Documents**

Source documents include original records and certified copies of original records of clinical findings, observations, or other activities in a clinical trial necessary for the reconstruction and evaluation of the trial. Examples of source documents include: medical histories, physical examination results, demographic data, subject diaries and pharmacy dispensing records.

Patient diary data will also serve as the CRF. No separate CRF will be created for this data.

**12.4 Data and Safety Monitoring Plan**

A comprehensive Data Safety and Monitoring Plan has been submitted by Northwestern University, has been approved by the DCP, and is on file there. Any future changes will be forwarded for review and approval.

**12.5 Sponsor or FDA Monitoring**

The NCI, DCP (or their designee), or FDA may monitor/audit various aspects of the study. These monitors will be given access to facilities, databases, supplies and records to review and verify data pertinent to the study.

**12.6 Record retention**

Clinical records for all participants, including CRFs, all source documentation (containing evidence to study eligibility, history and physical findings, laboratory data, results of consultations, etc.), as well as IRB records and other regulatory documentation will be retained by the Protocol Lead Investigator in a secure storage facility in compliance with HIPAA, OHRP, FDA regulations and guidances, and NCI/DCP requirements unless the standard at the site is more stringent. The records for all studies performed under an IND will be maintained, at a minimum, for two (2) years after the approval of a New Drug Application (NDA). For NCI/DCP, records will be retained for at least three (3) years after the completion of the research. NCI will be notified prior to the planned destruction of any materials. The records should be accessible for inspection and copying by authorized persons of the Food and Drug Administration.

**12.7 Cooperative Research and Development Agreement (CRADA)/Clinical Trials Agreement (CTA)**

All study agents are to be purchased commercially. No CRADA or CTA is applicable to this study. PEG will be supplied and packaged by NCI, DCP.

1. **STATISTICAL CONSIDERATIONS**

**13.1 Study Design/Endpoints**

This is a placebo controlled, three group 1:1:1 randomized study. The primary endpoint is treatment related changes in EGFR expression, namely EGFR (A)-EGFR (B), where A=after, B=before. Based upon the report by Roy et al., we hypothesize that 8g and 17g PEG 3350 will reduce EGFR expression by 21% and 30% respectively  Under this assumption we expect for “no change” group diff = 0, and for 21% change approximate reduction EGFR (A)-EGFR (B) ABOUT 37. Using pairwise t-test comparisons after one way ANOVA with 20 patients in each of the three groups, we have over 83% power to detect difference of d=37 points between one treatment group and the placebo group , when type 1 error is 0.02. The underlying assumption is that Corr [EGFR (A), EGFR (B)] = 0.5 and coefficients of variation are between 20% and 30%. We use PASS 2005 ([www.ncss.com](http://www.ncss.com/)) – see below. We will control for type 1 error using Bonferoni adjustment.

**Numeric Results for Two-Sample T-Test**

Null Hypothesis: Mean1=Mean2. Alternative Hypothesis: Mean1<Mean2

The standard deviations were assumed to be unknown and unequal.

**Power N1 N2 Ratio Alpha Beta Mean1 Mean2 S1 S2**

0.83522 20 20 1.000 0.02000 0.16478 138.000 175.000 40.000 35.000

**13.2 Sample Size/Accrual Rate**

Based on the power calculations presented in section 13.1, 60 patients must complete the study. To ensure that sufficient evaluable patients complete the study, 105 patients will be randomized into the study. It is estimated that approximately 2/3 of consented patients will pass screening. Therefore, 140 patients will be consented into the study to randomize 105 patients. A review of colonoscopies performed at NorthShore University HealthSystem and University of Chicago Hospital indicates that of the 200 procedures performed each week, 10-20% have a history of polyp or cancer. Approximately 50% of those will have some exclusion criteria or be logistically unavailable for recruitment. Therefore 10-20 eligible patients are seen per site per week. A conservative estimate of patient consent rate is 10-20%, yielding 1-4 patients per site per week. At 2 patients per week, the study will enroll 140 patients in 70 weeks (approximately 18 months).

**13.3 Randomization and Stratification**

Participant randomization is to be stratified by both recruitment site and number of ACFs at baseline. Since the primary outcome is a change in EGFR expression (A-B=post minus pre) same endoscopist will perform both exams for each individual patient.

Thus, the 4 strata will be 1) Evanston Hospital, 2) Glenbrook Hospital, 3) University of Chicago, and Boston University. It is difficult to predict the balance of recruited patients to each stratum. Patients will be randomized in blocks of 3 (PEG 17g, PEG 8g and placebo). Sufficient number of such blocks will be generated for all strata to accommodate the maximum number of patients that may be recruited.

**13.4 Primary Endpoint(s)**

The primary endpoint is described in section 8.1 and the statistics to be used are described in section 13.1.

- 1. **Secondary Endpoint(s)**

The secondary endpoints are described in section 8.2. For secondary endpoints, in animal studies we have observed treatment/control changes of 4.5 fold and 2.3 fold in apoptosis, 4 fold in proliferation. In addition, there will be a harvesting of 1-2 sigmoid ACF, if available, in the Before and in the After time point. Observed coefficients of variation were 0.1-0.6. Under similar effects and conditions for the secondary outcomes in the proposed studies, we will be able to detect most of underlying differences between treatment and control groups with 20 patients per group, with type 1 error of 1% or less, provided such differences truly exist. The Ki-67 and cleaved caspase 3 outcomes will be the number of cells positive over total number. The EGFR and SNAIL immunohistochemistry will be done by quantitation through chromovision and will be expressed as % of control (placebo, pre-treatment) The description and comparison of biomarkers in harvested ACF’s will be performed both with the view of Before – After possible change within each treatment group, as well as with the view of Treatment - Control groups for Before and for the After period. This will be done with the purpose of gaining knowledge of the range of changes to plan future studies in these markers. We anticipate about 20-25% of patients to be eligible for such harvesting.

**13.6 Reporting and Exclusions**

Compliance will be formally assessed on a monthly basis. Patients will be considered compliant if they have taken 80% of their study agent doses as directed. During the first month of study, patients will be counseled regarding the importance of taking all doses, but will not be removed from the study for missing doses, unless 10 consecutive days are missed. Patients who are found to be non-compliant at the monthly assessments will be kept in the study; their information will be used in the final ‘intent to treat’ analysis, which will contain all randomized patients.

**13.7 Evaluation of Toxicity**

All participants will be evaluable for toxicity from the time of their first dose of PEG 3350.

**13.8 Evaluation of Response**

All participants included in the study must be assessed for response to intervention, even if there are major protocol deviations or if they are ineligible.

All of the participants who met the eligibility criteria (with the possible exception of those who did not receive study agent) will be included in the main analysis. All conclusions regarding efficacy will be based on all eligible participants.

Sub-analyses may be performed on the subsets of participants, including those for whom major protocol deviations have been identified (e.g., early death due to other reasons, early discontinuation of intervention, major protocol violations, etc.). However, sub-analyses may not serve as the basis for drawing conclusions concerning efficacy. The reasons for excluding participants from the analysis should be clearly reported. For all measurements of response, observed average response in each group and the 95% confidence intervals will be provided.

**13.9 Interim Analysis**

No interim analyses are planned.

**13.10 Ancillary Studies**

No ancillary studies are planned.

1. **ETHICAL AND REGULATORY CONSIDERATIONS**
   1. **Form FDA 1572**

Prior to initiating this study, the Protocol Lead Investigator at the Lead or Participating Organization(s)will provide a signed Form FDA 1572 stating that the study will be conducted in compliance with regulations for clinical investigations and listing the investigators, at each site that will participate in the protocol.

- 1. **Other Required Documents**
     1. Signed and dated current (within two years) CV or biosketch for all investigators listed on the Form FDA 1572 for the Lead Organization and all Participating Organizations.
     2. Current medical licenses for all investigators listed on Form FDA 1572 for the Lead Organization and all Participating Organizations.
     3. Lab certification (e.g., CLIA, CAP) and lab normal ranges for all labs listed on Form FDA 1572 for the Lead Organization and all Participating Organizations.
     4. IRB Membership list/letter from IRB for the Lead Organization and all Participating Organizations.
     5. Documentation of training in “Protection of Human Research Subjects” for all investigators listed on the FDA Form 1572 for the Lead Organization and all Participating Organizations.
     6. Documentation of Federalwide Assurance number for the Lead Organization and all Participating Organizations.
     7. Signed receipt of Investigator Brochure
     8. Delegation of Responsibility form
  2. **Institutional Review Board Approval**

Prior to initiating the study and receiving agent, the Protocol Lead Investigator at the Lead Organization and/or the Participating Organization(s) must obtain written approval to conduct the study from the appropriate IRB. Should changes to the study become necessary, protocol amendments will be submitted to the DCP PIO according to DCP Amendment Guidelines. The DCP-approved amended protocol must be approved by the IRB prior to implementation.

- 1. **Informed Consent**

All potential study participants will be given a copy of the IRB-approved Informed Consent to review. The investigator will explain all aspects of the study in lay language and answer all questions regarding the study. If the participant decides to participate in the study, he/she will be asked to sign the Informed Consent document. The study agent(s) will not be released to a participant who has not signed the Informed Consent document. Subjects who refuse to participate or who withdraw from the study will be treated without prejudice.

Participants must be provided the option to allow the use of blood samples, other body fluids, and tissues obtained during testing, operative procedures, or other standard medical practices for further research purposes. A separate signature area is required to allow participants to opt out of allowing tissue to be used for further research.

The informed consent document must be reviewed and approved by NCI, DCP and the IRB at each Organization at which the protocol will be implemented prior to study initiation. Any subsequent changes to the informed consent must be approved by NCI, DCP and then submitted to each organization’s IRB for approval prior to initiation.

**14.5 Submission of Regulatory Documents**

All regulatory documents are collected by the Consortia Lead Organization and reviewed for completeness and accuracy. Once the Consortia Lead Organization has received complete and accurate documents from a participating organization, the Lead Organization will forward the regulatory documents to:

[Margaret](mailto:Margaret) Scheitrum, BS, CIP

CCS Associates, Inc.

1923 Landings Drive

Mountain View, CA 94043

mscheitrum@ccsainc.com

Regulatory documents that do not require an original signature may be sent electronically to the Lead Organization for review, which will then be forwarded to DCP electronically.

**14.6 Other**

This trial will be conducted in compliance with the protocol, Good Clinical Practice (GCP), and the applicable regulatory requirements.

1. **Financing, Expenses, and/or Insurance**

All research-related costs associated with participating in this study will be paid for and will not be the responsibility of the participant. However, it is possible that injury may result from participating in this study. Any expenses incurred as a result of research related injury will be the responsibility of the study participant and/or their insurance carrier.

**CONSENT FORM**

Polyethylene Glycol for Chemoprevention of Colon Carcinogenesis /DCP Protocol #: NWU 06-8-01

Principal Investigator (name): _______________ *[investigator’s name]*

Principal Investigator telephone number: ________________ *[telephone number]*

Sponsor (name): National Cancer Institute

**This is a clinical trial, a type of research study. Your study doctor will explain the clinical trial to you. This Consent Form gives information about the study. You are being given this information to help you decide if you would like to participate in the study. Clinical trials include only people who choose to take part. Your participation in this study is voluntary. If you do not wish to participate in this study, it will not affect the care you will receive. Please take your time to make your decision about taking part. You may discuss your decision with your friends and family. You can also discuss it with your health care team. If you have any questions you can ask your study doctor for more explanation.**

**EXPLANATION OF STUDY:**

**Nature and Purpose of Research Study:**

You are being asked to take part in this clinical research study because you are scheduled to undergo colonoscopy and have previously had colon polyps or cancer.

The purpose of this study is to find out what effects, good and/or bad, PEG (polyethylene glycol) has on you and your risk of developing colon cancer. Specifically, we want to find out if PEG may reduce the number of small abnormal changes in your colon that may lead to cancer.

**Study Drug Information:**

PEG (Polyethylene Glycol) can reduce the development of colon cancer in laboratory animals. PEG is widely used as a laxative medication for people who have constipation and is approved for this use by the FDA. The usual dose of PEG for people with constipation is 17g. In this study, some patients will receive 17g doses, some will receive 8g doses (approximately half-doses) and others will receive placebo (a powder that looks like PEG but does not contain PEG and is inactive). You will have a one in three chance of receiving a placebo on this study. Neither you, nor your physician, will know whether you are receiving PEG or placebo. "You will be “randomized” into one of the study groups. Randomization means that you are put into a group by chance. A computer program will place you in one of the study groups".

**Number of Participants:**

We plan to screen up to 140 people for this study.  105 eligible participants will be randomized and provided with study drug or placebo.

**INSERT purpose statement here:**

**The purpose of this project must be clearly defined in the following paragraph. Include background information on the study drug, device, or procedure (how does it work, why is the study needed, etc.). Include whether or not the drug/device is investigational or approved by the FDA (Food and Drug Administration). The duration of the subject’s involvement should be clearly stated.**

**Explanation of Procedures:**

If you are interested in participating in the study, you will need to have tests and procedures at the time of your colonoscopy to find out if you can be in the study.

**Before your colonoscopy, you will be asked:**

- Questions about your medical history and any medications you are taking.
- To have a physical exam during which your vital signs (such as heart rate and blood pressure) will be assessed.
- To have blood drawn (approximately 60ml or 4 tablespoons)
  - To make sure that your major organs are working correctly
  - For research purposes, and
  - To test for pregnancy, if you are female, and could be pregnant

**During your colonoscopy:**

- - - At least 6 and as many as 7 research biopsies will be taken in addition to those that would be taken if you were not on study. Biopsies will be taken using a tweezers-like device which is inserted through the colonoscope. Each biopsy will be less than ¼ inch in diameter.
    - To determine your eligibility for the study, your colon will also be inspected for small abnormal changes in your colon that may lead to cancer, called aberrant crypt foci (ACF).

**Within ten days after your colonoscopy:**

- The study doctor’s staff will call you to inform you if you are eligible to participate further in the study.
- The study doctor’s staff will instruct you on how to take the prescribed study medication. You will dissolve one packet of study medication in 8 ounces of water, juice, tea or coffee to drink once a day for six months

**During the six months you are taking study medication:**

- You will be asked to keep a daily diary of the following:
  - When you have taken your study medication
  - How many bowel movements you have had
  - If you are experiencing any discomfort
  - What medications you are taking
- If you experience constipation during the study, you will be asked to take only the additional laxatives provided by the study. You will be given tablets of bisacodyl (a laxative medication) to take if you require laxatives. Your study doctor will instruct you on when and how to take these laxatives. If you have been taking a stable dose of laxatives for the 90 days before your colonoscopy, you will be allowed to continue your current laxatives while participating in the study.
- You will be asked to limit your use of certain pain medication (such as aspirin, Tylenol and Advil) to no more than 30 total days in the 6 month (180 day) study. If you are taking aspirin for cardiac health, you will be allowed to continue taking aspirin at the same dose throughout the study.
- The doctor’s study staff will call you to check on your condition. You will be asked about your medications, bowel habits and any discomfort you may have experienced. If you are a woman, you will be asked if you suspect you might be pregnant.

**At the end of six months on the study medication:**

- You will be asked to return to the doctor’s office
- You will have a physical exam during which your vital signs (such as heart rate and blood pressure) will be assessed.
- You will have a blood draw to make sure that your major organs are working correctly and additional blood will be collected to be used for research. Approximately 60 ml or 4 tablespoons will be drawn.
- You will have a flexible sigmoidoscopy. A flexible sigmoidoscopy is an examination of the lower part of the colon or lower intestine. In this procedure, a tube is passed through the anus into the rectum and colon. This is similar to a colonoscopy; however, the tube is not inserted as far. The flexible sigmoidoscopy will take approximately 5-10 minutes and you will be able to drive yourself home after the examination.
  - To clear out the lower part of your colon you will be administered a Fleets enema.
  - Between 6 and 9 biopsies of the lining of the rectum and colon (each less than ¼ inch in diameter) will be taken using a tweezer-like device inserted through the scope.
  - Your colon will also be inspected for abnormal crypt foci (ACF). The number of ACF present in your colon at the 6 month visit, if any, will be compared to the number present at your colonoscopy at the beginning of the study.

**One month after you finish the study:**

- The doctor’s study staff will contact you by telephone to check on your medical condition. If any of the laboratory tests, or examinations of your colon reveal any abnormalities that could indicate that you have a medical condition, the study staff will let you know and give you further instructions.

**Schedule of Events:**

| Day | Events |
| --- | --- |
| Scheduled Colonoscopy | - Sign informed consent - Review medical history with study staff - Receive a physical exam - Have blood drawn for lab tests and research - Have a colonoscopy with ACF count and biopsies |
| 6-10 days after your colonoscopy | - Start study medication (PEG or placebo) - Begin keeping daily diary |
| Weeks 1 and 2, and Months 1, 2, 3, 4, and 5 after starting study drug | - Telephone contact with study staff - Review health status and diary data |
| Six Months on study drug | - Doctor’s office visit - Receive physical exam - Review health status and diary data - Have blood drawn for lab tests and research - Have a flexible sigmoidoscopy with ACF count and biopsies |
| One Month after finishing study drug | - Telephone contact with study staff - Review health status |

**The subject’s involvement should be defined here. Insert in this section information about whether this is a blinded study or if there is randomization. Special issues with the study design should also be described here. Examples: This is a Phase I study, this is a dose escalation study, or this is a study that has a roll-in phase. All information must be presented in such a way as to ensure that the subject has complete understanding of what will be happening in the study.**

**Lay language is extremely important in this section. Subjects must understand all procedures that will take place during the course of the research. It is preferable to minimize technical/medical terminology if possible.**

**Alternative Therapy:**

The alternative is not to participate in this study. You may be eligible for other colon cancer prevention studies. The standard approach for people with prior history of colon polyps is to go through with your scheduled colonoscopy.

If you do not wish to participate in this study, proceed with your regularly scheduled colonoscopy and repeat the test as determined by you and your doctor. ALTERNATIVE THERAPY (examples of statements

Investigators should be reasonably specific about describing the nature and type of available alternatives. It is not sufficient simply to state that "the researcher will discuss alternative treatments" with the subject.

**POSSIBLE BENEFITS:**

You may not benefit from this research study. If PEG is effective in preventing colon cancer; you may receive some benefit while you are taking the medication. It is not known whether any possible benefit would persist when the treatment is stopped. We will not be able to tell from this study whether your personal risk of developing colon cancer has been lowered. Your participation in this study may aid our ability to develop a new drug to prevent colon cancer.

**RISKS AND DISCOMFORTS:**

Your participation in this study may involve the following risks. We cannot predict all risks or side effects. There may be additional risks from the study treatment that are not known.

1. Blood Draws

Possible side effects from blood drawing include bruising, local discomfort and infection. Fewer than 1 % (one in one hundred) people who have their blood drawn will develop an infection.

2. PEG

Nausea, abdominal cramping, or gas may occur. In studies of constipated patients, these occurred in up to 11% of patients. If any of these effects persist or worsen, notify your study doctor/ staff promptly. Many people using this medication do not have serious side effects. Inform your study doctor/staff immediately if you have excessive number of bowel movements and persistent [diarrhea](http://www.medicinenet.com/script/main/art.asp?articlekey=1900) while using this medication. In rare instances, people are allergic to PEG. Symptoms of a serious allergic reaction may include: [rash](http://www.medicinenet.com/script/main/art.asp?articlekey=1992), itching, swelling, severe dizziness, trouble breathing. If you notice other effects not listed above, contact your study doctor or staff.

3. Biopsies of the intestine:

Bleeding can occur from biopsies or the removal of polyps, but it is usually minimal and stops quickly or can be controlled. Major bleeding episodes that require blood transfusions or hospitalization are extremely rare.

4. Flexible Sigmoidoscopy:

Flexible Sigmoidoscopy is generally a safe test although rare complications can occur. These can include some mild pain or discomfort, like a feeling of fullness, felt during this test. Even more rarely (less than 1 in 10,000 times), a hole can be made in the side of the rectum or colon that can require abdominal surgery.

5. Placebo Risks:

Participants assigned to the placebo group will receive placebo (a powder which does not have any active ingredient) instead of active study medication (which is PEG) for the duration of the study. The risks of receiving placebo are the same as not receiving any treatment for your condition. Any concerns you may have about this should be discussed with the study doctor.

**REPRODUCTIVE RISKS:**

You should not become pregnant while on this study, because the drugs in this study may affect an unborn baby. Women should not breastfeed a baby while on this study. It is important you understand that if you could become pregnant, you need to use birth control while on this study. You will not be eligible to continue participating in this study if you are, or plan to become pregnant.

Risks should be categorized according to probability. Risks that are very probable should be identified as such, while it should be stated that other risks are rare or unlikely. An example of a classification system that you may want to use is as follows:

Very Common 10% or more

Common (frequent) 1 to <10%

Uncommon (infrequent) 0.1 to <1%

Rare 0.01 to <0.1%

Very Rare <0.01%

(From the CIOMS {Council for International Organization of Medical Sciences}

All reasonably foreseeable risks, discomforts, inconvenience, and harms that are associated with the research activity should be described.

If additional risks are identified during the course of the research, the consent process and documentation will require revisions to inform subjects as they are recontacted or newly contacted.

**EXPLANATION OF INVESTIGATOR'S AVAILABILITY TO ANSWER QUESTIONS:**

The study doctor will answer any questions you have. Any new information or change in the study will be given to you as soon as it becomes available.

**CONFIDENTIALITY:**

Involvement in this research study may result in a loss of privacy, since persons other than the investigator(s) might view your study records. Some of your health information, and information about your specimen, from this study may be sent to a central database for research. Your name or contact information will not be put in the database. Unless required by law the following people can review your study records:

- Study Investigators
- Study Investigators’ staff
- Representatives of the National Cancer Institute ( NCI) or the US Food and Drug Administration (FDA) under data collection authority Title 42 U.S.C. 285
- The Institutional Review Board

All of these individuals are required to keep your personal information confidential.

Results of this study may be used for research, presented at research meetings, and/ or published in scientific journals. Under all of these circumstances, your identity will be protected.

**COMPENSATION DISCLAIMER:** Choose ONE of the following three statements. Delete the other two statements you do not want. Please note that no changes are permitted in this section.

1If you become hurt or sick because of being in this research study, you can get medical treatment at *________________________* [*name of medical center*]. You or your health insurance plan will be billed. No money has been set aside to pay the costs of this treatment. You can ask for more information from the *________________________* [*name of research center*].

**PAYMENT FOR PARTICIPATION:**

To offset your costs of participating in this study, you will be reimbursed a total of $350 according to the following schedule:

- $50 after the initial visit
- $50 after the 1 month clinic visit
- $250 after the 6 month clinic visit.

If you do not complete the research study, you will only be paid for the length of time that you were a subject. You will be paid at the time you withdraw from the study.

**ADDITIONAL COSTS:**

There is expected to be no additional cost to you from being in this research study.

**EXPLANATION OF ABILITY TO WITHDRAW FROM STUDY:**

Your participation in this research study is voluntary and you are free to withdraw at any time. Choosing not to participate or withdrawing from this study will not affect your present or future treatment to which you are otherwise entitled. Your doctor may stop this study or take you out of the study without your permission.

**YOUR RIGHTS AS A RESEARCH SUBJECT:**

You may get more information about your rights from the Chairperson of the Institutional Review Board (IRB). You can also call the IRB Coordinators at __________________ *[telephone number].* These are the people you should contact about any problems or research-related injuries that happen during the research study.

**WHERE CAN I GET MORE INFORMATION?**

The National Cancer Institute will obtain information from this clinical trial under data collection authority Title 42 U.S.C. 285.

You may visit the NCI website at http://cancer.gov/ for more information about studies or general information about cancer. You may also call the NCI Cancer Information Service to get the same information at: 1-800-4-CANCER (1-800-422-6237).

A description of this clinical trial will be available on http://www.ClinicalTrials.gov, if required by US law. This website will not include information that can identify you. At most, the website will include a summary of the results. You can search this website at any time.

********************************************************************************

**INDIVIDUAL PROVIDING EXPLANATION:**

The procedures and/or investigations described in the above paragraphs have been explained to you by (print):

| **Name of Person Explaining Study** | **(PRINT)** |
| --- | --- |
| **Signature of Person Explaining Study** | *(Sign)* |
| **Date Signed** | (Date) |

If you have additional questions at any time during the study, you may contact your study doctor __________________ *[name(s)]* at __________________ *[telephone number].* Insert a local telephone number or toll free number.

**CONSENT TO PARTICIPATE:**

I understand that __________________ *[investigator’s name(s)],* and his/her assistants will supervise the study. I have read this consent form or have had it read to me. I understand what will happen if I enroll in this research study. I understand the possible benefits and risks of the study. I have been told about all of my treatment options. I give permission for the research study procedures described in this consent form.

I have reviewed this information with the study director and/or staff. I have had enough time with the study director and/or staff to talk about all of my questions and concerns. I willingly consent to be a part of this study. I will receive a signed and dated copy of this Consent Form.

| **Subject’s Name**  **(Please PRINT)** |  |
| --- | --- |
| **Subject’s Signature** |  |
| **Date Signed** |  |
| **Witness' Signature** |  |
| **Date Signed** |  |
| **Physician’s Signature/ Investigator Signature** |  |
| **Date Signed** |  |
| **Legal Representative Signature** | **Delete if not required by circumstances** |
| **Relationship to Subject or other basis for legal authority** |  |
| **Date Signed** |  |

Witness signature = witness the signature of the subject and/or witness the consent process.

**CONSENT FORM**

**CONSENT FOR THE STORAGE AND FUTURE (UNSPECIFIED) TESTING**

**OF YOUR SAMPLES Obtained from the study:**

Polyethylene Glycol for Chemoprevention of Colon Carcinogenesis /DCP Protocol #: NWU 06-8-01

Principal Investigator: __________________ *[investigator’s name(s)],*

Principal Investigator telephone number: __________________ *[telephone number].* Sponsor: National Cancer Institute

**EXPLANATION OF STUDY:**

Nature and Purpose of Research Study: As part of the ongoing scientific and research activities, you are being asked to allow some of the blood and biopsy, samples that are left over to be used for unspecified future research. If you agree, the leftover samples will be kept and may be used in research to learn more about cancer or other diseases.

The samples will only be given to researchers approved by NorthShore University HealthSystem and any future research done will have to be first approved by the researcher’s Institutional Review Board (these are research oversight agencies interested in protecting the rights of research subjects and ethical issues).

Reports about the future research done with your samples will not be given to you or your study doctor because the research will not have an effect on your care. These samples may be stored for a long time, even after your death. These future research studies may also include genetic research (about diseases that are passed on in families). Even if your samples are used for this kind of research, the results will not be put in your health records. You do not have to participate in this study in order to participate in the main study.

This Consent Form gives information about the study that you will be able to discuss with your doctor. You are being given this information to help you decide if you would like to participate. If you have any questions, you can ask the study doctor and/or staff. INSERT purpose statement here:

The purpose of this project must be clearly defined in the following paragraph. Include background information on the study drug, device, or procedure (how does it work, why is the study needed, etc.). Include whether or not the drug/device is investigational or approved by the FDA (Food and Drug Administration). The duration of the subject’s involvement should be clearly stated.

Explanation of Procedures:

This research involves only samples that will have already been taken during the course of your participation in the study.

The subject’s involvement should be defined here. Insert in this section information about whether this is a blinded study or if there is randomization. Special issues with the study design should also be described here. Examples: This is a Phase I study, this is a dose escalation study, or this is a study that has a roll-in phase. All information must be presented in such a way as to ensure that the subject has complete understanding of what will be happening in the study.

Lay language is extremely important in this section. Subjects must understand all procedures that will take place during the course of the research. It is preferable to minimize technical/medical terminology if possible.

Alternative Therapy:

You may choose not to allow for storage and future testing of your samples. Your decision whether or not to allow for this storage and future testing will have no Impact on your participation in the main protocol, Polyethylene Glycol for Chemoprevention of Colon Carcinogenesis /DCP Protocol #: NWU 06-8-01

Investigators should be reasonably specific about describing the nature and type of available alternatives. It is not sufficient simply to state that "the researcher will discuss alternative treatments" with the subject.

**POSSIBLE BENEFITS:**

There will be no direct benefit to you by your participation in this research. However, your participation may help learn more about what causes cancer and other diseases, how to prevent them, how to treat them, and how to cure them

**RISKS AND DISCOMFORTS:**

Your participation in this study does not involve any physical risk to you. Uncommon (infrequent) 0.1 to <1% Rare 0.01 to <0.1%

Very Rare <0.01%

(From the CIOMS {Council for International Organization of Medical Sciences}

All reasonably foreseeable risks, discomforts, inconvenience, and harms that are associated with the research activity should be described.

If additional risks are identified during the course of the research, the consent process and documentation will require revisions to inform subjects as they are recontacted or newly contacted.

**EXPLANATION OF INVESTIGATOR'S AVAILABILITY TO ANSWER QUESTIONS:**

The study doctor will answer any questions you have based on current medical knowledge. Any new information or changes in the study that may affect your health or your willingness to continue in the study will be given to you as soon as they become available.

**CONFIDENTIALITY:**

If information from this study is published, presented at meetings or placed in a report, your name and other personal information will not be used. Every effort will be made to keep your personal medical information confidential. Your study related information may be examined by other researchers in this study, by the sponsoring organization, by the ________________________ *[name of center]* Institutional Review Board, or by the Food and Drug Administration (FDA).

The regulations require that subjects be told the extent to which their personally identifiable, private information will be held in confidence. Blanket access to patient medical records defeats confidentiality. State that only study-related records or research-related records may be reviewed by others involved in the research (such as the sponsor, co-investigators, and the IRB).

**COMPENSATION DISCLAIMER: Choose ONE of the following three statements. Delete the other two statements you do not want. Please note that no changes are permitted in this section.**

1)If you have an injury or illness because of being in this research study, you can obtain medical treatment at *________________________* [*name of medical center*]. You and/or your health plan or insurance company will be billed since no funds have been set aside to pay the costs of this treatment. You can obtain more information from the *________________________* *[name of research center]*.2)

If research-related injury (i.e., physical, psychological, social, financial, or otherwise) is possible in research that is more than minimal risk, an explanation must be given of whatever voluntary compensation and treatment will be provided. Note that the regulations do not limit injury to "physical injury." This is a common misinterpretation.

The regulations prohibit

(i) requiring subjects to waive any of their legal rights, and

(ii) leading subjects to believe they are waiving their rights. Consent language regarding compensation for injury must be selected carefully so that subjects are not given the impression that they have no recourse to seek satisfaction beyond the institution's voluntarily chosen limits.

**PAYMENT FOR PARTICIPATION:**

You will not be paid for your participation in this project.

**ADDITIONAL COSTS:**

There is expected to be no additional cost to you from participating in this research study.

**EXPLANATION OF ABILITY TO WITHDRAW FROM STUDY:**

Your participation in this research is voluntary. Your decision whether or not to allow for testing of your samples will in no way affect your present or future medical treatment. If you decide now that your samples can be kept for this research, you can change your mind at any time. Just contact your study doctor and let him or her know that you do not want us to use your samples. Then any tissue that remains will no longer be used for research.

**YOUR RIGHTS AS A RESEARCH SUBJECT:**

You may obtain additional information about your rights as a research subject from the Chairperson of the Institutional Review Board (IRB) or the IRB Coordinators. The telephone number is __________________ *[telephone number].* These are the individuals to whom you should report any problems or injuries that may be due to the research study.

Please read each sentence and think about your choice. After reading each sentence, please initial your choice. You can participate in the treatment part of the study without participating in all or part of the research studies.

| ._____ I do agree my blood and tissue specimens may be kept for use in research to learn about, prevent, treat, or cure cancer.  _____ I do NOT agree my blood and tissue specimens may be kept for use in research to learn about, prevent, treat, or cure cancer. |
| --- |
| _____ I do agree my blood and tissue specimens may be kept for research about other health problems (for example: causes of diabetes, Alzheimer's disease, and heart disease).  _____ I do NOT agree my blood and tissue specimens may be kept for research about other health problems (for example: causes of diabetes, Alzheimer's disease, and heart disease). |

**INDIVIDUAL PROVIDING EXPLANATION:**

The procedures and/or investigations described in the above paragraphs have been explained to you by (print):

| Name of Person Explaining Study | (PRINT) |
| --- | --- |
| Signature of Person Explaining Study | (Sign) |
| Date Signed | (Date) |

If you have additional questions at any time during the study, you may contact __________________ *[name(s)]* at __________________ *[telephone number].* Insert a local telephone number or toll free number.

**CONSENT TO PARTICIPATE:**

I understand that the activities will be supervised by __________________ *[investigator’s name(s)],* FILL IN NAMand whomever he/she may designate as his/her assistant(s). I have read this consent form or have had it read to me. I have been informed of the nature and purposes of the activities, treatment, the possible risks and discomforts, the possible benefits and the possible alternative methods of treatment. I hereby authorize the performance of the activities described in this consent form.

I have read and discussed the explanation of this study with the study director and/or staff I have had enough time with the study director and/or staff to discuss all of my questions and concerns. I willingly consent to be a part of this study. I will receive a signed and dated copy of this Consent Form.

| **Subject’s Name (Please PRINT)** |  |
| --- | --- |
| **Subject’s Signature** |  |
| **Date Signed** |  |
| **Witness' Signature** |  |
| **Date Signed** |  |
| **Physician’s Signature/ Investigator Signature** |  |
| **Date Signed** |  |

Witness signature = witness the signature of the subject and/or witness the consent process.

Physician signature = not required, but if using implies that the physician was a part of the consent process; therefore, the signature must be obtained on the same date as the subject.

Physician signature = not required, but if using implies that the physician was a part of the consent process; therefore, the signature must be obtained on the same date as the subject.

Legal representative signature = only required in cases of mental incompetency on the part of the subject. Use of a legal representative implies that this person was a part of the consent process; therefore, if both the subject and the representative sign the consent form, the signatures must be obtained on the same date.

REMINDER: Once approved, all blank spaces must be completed or the consent form will be considered invalid and the subject will not be enrolled.

# APPENDIX A - Performance Status Criteria

| **ECOG Performance Status Scale** | | **Karnofsky Performance Scale** | |
| --- | --- | --- | --- |
| Grade | Descriptions | Percent | Description |
| 0 | Normal activity. Fully active, able to carry on all pre-disease performance without restriction. | 100 | Normal, no complaints, no evidence of disease. |
|  |  | 90 | Able to carry on normal activity; minor signs or symptoms of disease. |
| 1 | Symptoms, but ambulatory. Restricted in physically strenuous activity, but ambulatory and able to carry out work of a light or sedentary nature (e.g., light housework, office work). | 80 | Normal activity with effort; some signs or symptoms of disease. |
|  |  | 70 | Cares for self, unable to carry on normal activity or to do active work. |
| 2 | In bed <50% of the time. Ambulatory and capable of all self-care, but unable to carry out any work activities. Up and about more than 50% of waking hours. | 60 | Requires occasional assistance, but is able to care for most of his/her needs. |
|  |  | 50 | Requires considerable assistance and frequent medical care. |
| 3 | In bed >50% of the time. Capable of only limited self-care, confined to bed or chair more than 50% of waking hours. | 40 | Disabled, requires special care and assistance. |
|  |  | 30 | Severely disabled, hospitalization indicated. Death not imminent. |
| 4 | 100% bedridden. Completely disabled. Cannot carry on any self-care. Totally confined to bed or chair. | 20 | Very sick, hospitalization indicated. Death not imminent. |
|  |  | 10 | Moribund, fatal processes progressing rapidly. |
| 5 | Dead. | 0 | Dead. |

REMINDER: Once approved, all blank spaces must be completed or the consent form will be considered invalid and the subject will not be enrolled.

**Appendix B - Patient Diary**

**Patient ID#:** __ __ __ __ __ __ __

**1. Date:** __ __/__ __/__ __ __ __ (mm/dd/yyyy)

🞎 Sunday 🞎 Monday 🞎 Tuesday 🞎 Wednesday 🞎 Thursday 🞎 Friday 🞎 Saturday

**2. Time study drug taken:** __ __:__ __ 🞎 am 🞎pm or 🞎 No dose taken

**3. Total number of stools today: __ __**

**4. Did you feel you had to rush to the bathroom?**

🞎 Yes 🞎 No

**5. Stool consistency (on average):**

🞎 Hard 🞎 Formed 🞎 Soft 🞎 Watery

**6. Abdominal pain (on average):**

🞎 None 🞎 Mild 🞎 Moderate 🞎 Severe

**7. Bloating (on average):**

🞎 None 🞎 Mild 🞎 Moderate 🞎 Severe

**8. Flatulence (on average):**

🞎 None 🞎 Mild 🞎 Moderate 🞎 Severe

**9. Overall today, how would you rate your bowel habits?**

🞎 Very good 🞎 Good 🞎 Fair 🞎 Poor 🞎 Very poor

**10. Laxatives used today:** 🞎 None 🞎 10 mg bisacodyl 🞎 Other, specify: ______________

**11. Have you had any health problems today?** 🞎 No 🞎Yes

If yes, please describe: ________________________________________________________________________

_______________________________________________________________________

_______________________________________________________________________

**12. Have you had any changes in your medications? 🞎 No 🞎Yes**

If yes, what new medications are you taking: ______________________________________________________

______________________________________________________

**Appendix C - Victoria Bowel Performance Scale**

**
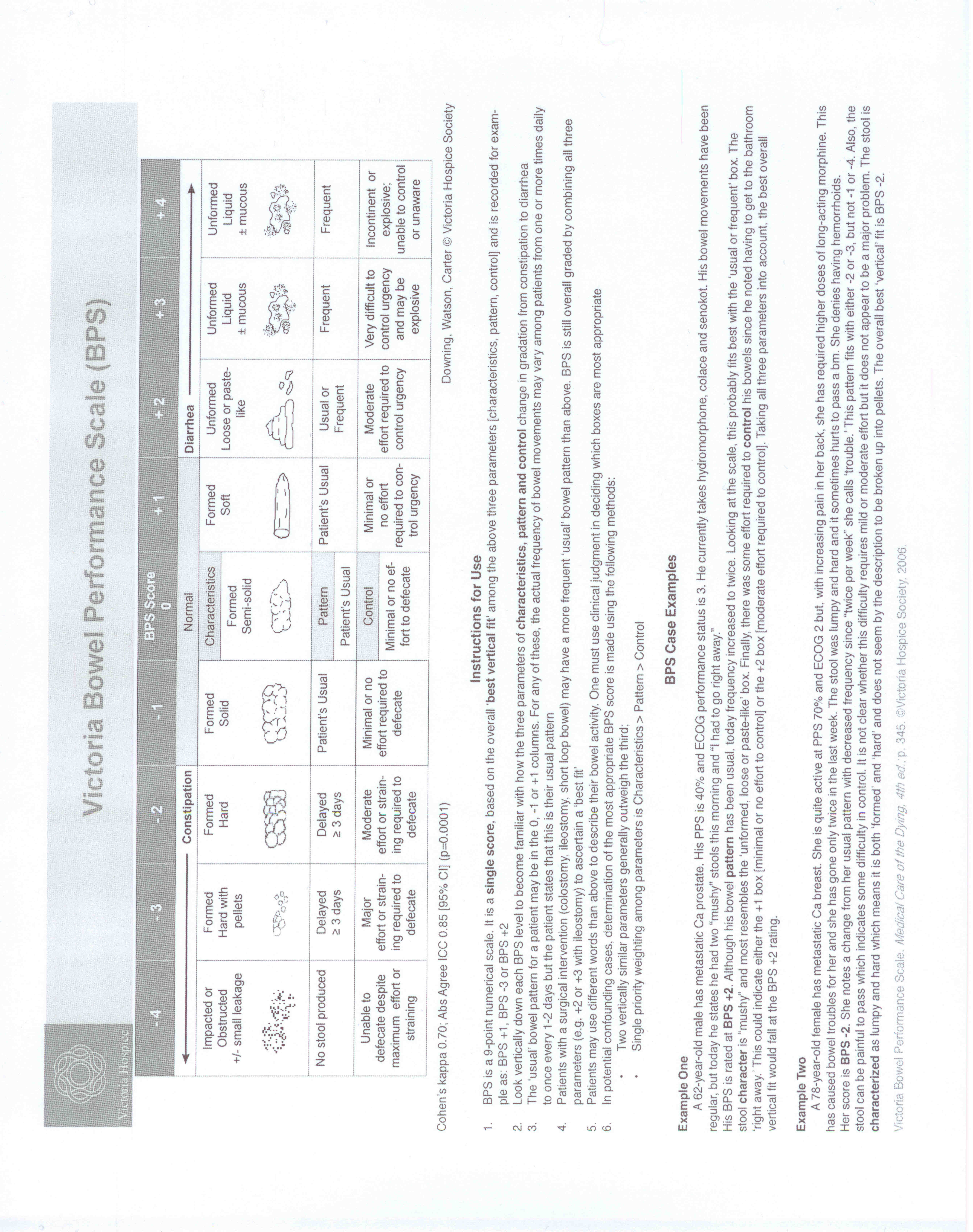
**

**REFERENCES**

1. Stevens RG, Swede H, Rosenberg DW. Epidemiology of colonic aberrant crypt foci: Review and analysis of existing studies. Cancer Lett 2006.

**2. Takayama T, Miyanishi K, Hayashi T, Kukitsu T, Takanashi K, Ishiwatari H, et al. Aberrant crypt foci: detection, gene abnormalities, and clinical usefulness. Clin Gastroenterol Hepatol 2005;3:S42-5.**

**3. Jemal A, Siegel R, Ward E, Murray T, Xu J, Thun MJ. Cancer statistics, 2007. CA Cancer J Clin 2007;57:43-66.**

**4. Jemal A, Murray T, Ward E, Samuels A, Tiwari RC, Ghafoor A, et al. Cancer statistics, 2005. CA Cancer J Clin 2005;55:10-30.**

**5. Walsh JM, Terdiman JP. Colorectal cancer screening: clinical applications. Jama 2003;289:1297-302.**

**6. Walsh JM, Terdiman JP. Colorectal cancer screening: scientific review. Jama 2003;289:1288-96.**

**7. Zack DL, DiBaise JK, Quigley EM, Roy HK. Colorectal cancer screening compliance by medicine residents: perceived and actual. Am J Gastroenterol 2001;96:3004-8.**

**8. Kim YI. AGA technical review: impact of dietary fiber on colon cancer occurrence. Gastroenterology 2000;118:1235-57.**

**9. Alberts DS, Martinez ME, Roe DJ, Guillen-Rodriguez JM, Marshall JR, van Leeuwen JB, et al. Lack of effect of a high-fiber cereal supplement on the recurrence of colorectal adenomas. Phoenix Colon Cancer Prevention Physicians' Network. N Engl J Med 2000(342):1156-62.**

**10. Schatzkin A, Lanza E, Corle D, Lance P, Iber F, Caan B, et al. Lack of effect of a low-fat, high-fiber diet on the recurrence of colorectal adenomas. Polyp Prevention Trial Study Group. N Engl J Med 2000;342:1149-55.**

**11. Earnest DL, Holubec H, Wali RK, Jolley CS, Bissonette M, Bhattacharyya AK, et al. Chemoprevention of azoxymethane-induced colonic carcinogenesis by supplemental dietary ursodeoxycholic acid. Cancer Res 1994;54:5071-4.**

**12. Seraj MJ, Umemoto A, Kajikawa A, Mimura S, Kinouchi T, Ohnishi Y, et al. Effects of dietary bile acids on formation of azoxymethane-induced aberrant crypt foci in F344 rats. Cancer Lett 1997;115:97-103.**

**13. Alberts DS, Martinez ME, Hess LM, Einspahr JG, Green SB, Bhattacharyya AK, et al. Phase III trial of ursodeoxycholic acid to prevent colorectal adenoma recurrence. J Natl Cancer Inst 2005;97:846-53.**

**14. Corpet DE, Tache S. Most effective colon cancer chemopreventive agents in rats: a systematic review of aberrant crypt foci and tumor data, ranked by potency. Nutr Cancer 2002;43:1-21.**

**15. Roy HK, Gulizia J, DiBaise JK, Karolski WJ, Ansari S, Madugula M, et al. Polyethylene glycol inhibits intestinal neoplasia and induces epithelial apoptosis in Apc(min) mice. Cancer Lett 2004;215:35-42.**

**16. Roy HK, Kunte DP, Koetsier JL, Hart J, Kim YL, Liu Y, et al. Chemoprevention of colon carcinogenesis by polyethylene glycol: Suppression of epithelial proliferation via modulation of snail/ -catenin signaling. Mol Cancer Ther 2006;5:2060-2069.**

**17. DiPalma JA, DeRidder PH, Orlando RC, Kolts BE, Cleveland MB. A randomized, placebo-controlled, multicenter study of the safety and efficacy of a new polyethylene glycol laxative. Am J Gastroenterol 2000;95:446-50.**

**18. Chaussade S. Mechanisms of action of low doses of polyethylene glycol in the treatment of functional constipation. Ital J Gastroenterol Hepatol 1999;31 Suppl 3:S242-4.**

**19. Tache S, Parnaud G, Van Beek E, Corpet DE. Polyethylene glycol, unique among laxatives, suppresses aberrant crypt foci, by elimination of cells. Scand J Gastroenterol 2006;41:730-6.**

**20. Roberts MC, Millikan RC, Galanko JA, Martin C, Sandler RS. Constipation, laxative use, and colon cancer in a North Carolina population. Am J Gastroenterol 2003;98:857-64.**

**21. Arber N, Levin B. Chemoprevention of colorectal cancer: ready for routine use? Curr Top Med Chem 2005;5:517-25.**

**22. Baron JA, Cole BF, Sandler RS, Haile RW, Ahnen D, Bresalier R, et al. A randomized trial of aspirin to prevent colorectal adenomas. N Engl J Med 2003;348: 891-9.**

**23. Sandler RS, Halabi S, Baron JA, Budinger S, Paskett E, Keresztes R, et al. A randomized trial of aspirin to prevent colorectal adenomas in patients with previous colorectal cancer. N Engl J Med 2003;348:883-90.**

**24. Ladabaum U, Chopra CL, Huang G, Scheiman JM, Chernew ME, Fendrick AM. Aspirin as an adjunct to screening for prevention of sporadic colorectal cancer. A cost-effectiveness analysis. Ann Intern Med 2001;135:769-81.**

**25. Suleiman S, Rex DK, Sonnenberg A. Chemoprevention of colorectal cancer by aspirin: a cost-effectiveness analysis. Gastroenterology 2002;122:78-84.**

**26. Graumlich JF. Preventing gastrointestinal complications of NSAIDs. Risk factors, recent advances, and latest strategies. Postgrad Med 2001;109:117-20, 123-8.**

**27. Nadir Arber MD, Craig J. Eagle MD, Julius Spicak MD, István Rácz MD, Petr Dite MD, Jan Hajer MD, et al. Celecoxib for the Prevention of Colorectal Adenomatous Polyps. NEJM 2006;355:885-895.**

**28. Herendeen JM, Lindley C. Use of NSAIDs for the chemoprevention of colorectal cancer. Ann Pharmacother 2003;37:1664-74.**

**29. Hippisley-Cox J, Coupland C. Risk of myocardial infarction in patients taking cyclo-oxygenase-2 inhibitors or conventional non-steroidal anti-inflammatory drugs: population based nested case-control analysis. Bmj 2005;330:1366.**

**30. Mukherjee D, Nissen SE, Topol EJ. Risk of cardiovascular events associated with selective COX-2 inhibitors. Jama 2001;286:954-9.**

**31. Monica M. Bertagnolli MD, Craig J. Eagle MD, Ann G. Zauber PD, Mark Redston MD, Scott D. Solomon MD, KyungMann Kim PD, et al. Celecoxib for the Prevention of Sporadic Colorectal Adenomas. NEJM 2006;355:873-884.**

**32. Bresalier RS, Sandler RS, Quan H, Bolognese JA, Oxenius B, Horgan K, et al. Cardiovascular events associated with rofecoxib in a colorectal adenoma chemoprevention trial. N Engl J Med 2005;352:1092-102.**

**33. Stoner GD, Budd GT, Ganapathi R, DeYoung B, Kresty LA, Nitert M, et al. Sulindac sulfone induced regression of rectal polyps in patients with familial adenomatous polyposis. Adv Exp Med Biol 1999;470:45-53.**

**34. Rossouw JE, Anderson GL, Prentice RL, LaCroix AZ, Kooperberg C, Stefanick ML, et al. Risks and benefits of estrogen plus progestin in healthy postmenopausal women: principal results From the Women's Health Initiative randomized controlled trial. Jama 2002;288:321-33.**

**35. Serrano D, Lazzeroni M, Decensi A. Chemoprevention of colorectal cancer: an update. Tech Coloproctol 2004;8 Suppl 2:s248-52.**

**36. Poynter JN, Gruber SB, Higgins PD, Almog R, Bonner JD, Rennert HS, et al. Statins and the risk of colorectal cancer. N Engl J Med 2005;352:2184-92.**

**37. Jacobs EJ, Rodriguez C, Brady KA, Connell CJ, Thun MJ, Calle EE. Cholesterol-lowering drugs and colorectal cancer incidence in a large United States cohort. J Natl Cancer Inst 2006;98:69-72.**

**38. Asano T, McLeod RS. Dietary fibre for the prevention of colorectal adenomas and carcinomas. Cochrane Database Syst Rev 2002.:CD003430.**

**39. Weingarten MA, Zalmanovici A, Yaphe J. Dietary calcium supplementation for preventing colorectal cancer and adenomatous polyps. Cochrane Database Syst Rev 2004:CD003548.**

**40. Grau MV, Rees JR, Baron JA. Chemoprevention in gastrointestinal cancers: current status. Basic Clin Pharmacol Toxicol 2006;98:281-7.**

**41. Cole BF, Baron JA, Sandler RS, Haile RW, Ahnen DJ, Bresalier RS, et al. Folic acid for the prevention of colorectal adenomas: a randomized clinical trial. Jama 2007;297:2351-9.**

**42. Parnaud G, Tache S, Peiffer G, Corpet DE. Polyethylene-glycol suppresses colon cancer and causes dose-dependent regression of azoxymethane-induced aberrant crypt foci in rats. Cancer Res 1999;59:5143-7.**

**43. Dipalma JA, Cleveland MV, McGowan J, Herrera JL. A randomized, multicenter, placebo-controlled trial of polyethylene glycol laxative for chronic treatment of chronic constipation. Am J Gastroenterol 2007;102:1436-41.**

**44. Corpet DE, Parnaud G, Delverdier M, Peiffer G, Tache S. Consistent and fast inhibition of colon carcinogenesis by polyethylene glycol in mice and rats given various carcinogens. Cancer Res 2000;60:3160-4.**

**45. Shpitz B, Klein E, Buklan G, Neufeld D, Nissan A, Freund HR, et al. Suppressive effect of aspirin on aberrant crypt foci in patients with colorectal cancer. Gut 2003;52:Shpitz, B., Klein, E., Buklan, G., Neufeld, D., Nissan, A., Freund, H. R., Grankin, M., and Bernheim, J. Suppressive effect of aspirin on aberrant crypt foci in patients with colorectal cancer. Gut, 52: 1598-601.**

**46. Takayama T, Katsuki S, Takahashi Y, Ohi M, Nojiri S, Sakamaki S, et al. Aberrant crypt foci of the colon as precursors of adenoma and cancer. N Engl J Med 1998;339:1277-84.**

**47. Roy HK, Karolski WJ, Ratashak A. Distal bowel selectivity in the chemoprevention of experimental colon carcinogenesis by the non-steroidal anti-inflammatory drug nabumetone. Int J Cancer 2001;92:609-15.**

**48. Dorval E, Jankowski JM, Barbieux JP, Viguier J, Bertrand P, Brondin B, et al. Polyethylene glycol and prevalence of colorectal adenomas. Gastroenterol Clin Biol 2006;30:1196-9.**

**49. Roy HK, DiBaise JK, Black J, Karolski WJ, Ratashak A, Ansari S. Polyethylene glycol induces apoptosis in HT-29 cells: potential mechanism for chemoprevention of colon cancer. FEBS Lett 2001;496:143-6.**

**50. Parnaud G, Corpet DE, Gamet-Payrastre L. Cytostatic effect of polyethylene glycol on human colonic adenocarcinoma cells. Int J Cancer 2001;92:63-9.**

**51. Bienz M, Clevers H. Linking colorectal cancer to Wnt signaling. Cell 2000;103:311-20.**

**52. Oshima M, Murai N, Kargman S, Arguello M, Luk P, Kwong E, et al. Chemoprevention of intestinal polyposis in the Apcdelta716 mouse by rofecoxib, a specific cyclooxygenase-2 inhibitor. Cancer Res 2001;61:1733-40.**

**53. Palmer HG, Larriba MJ, Garcia JM, Ordonez-Moran P, Pena C, Peiro S, et al. The transcription factor SNAIL represses vitamin D receptor expression and responsiveness in human colon cancer. Nat Med 2004;10:917-9.**

**54. Roy HK, Smyrk, T. C., Koetsier, J., Victor, T. A., and Wali, R. K. The transcriptional repressor SNAIL is overexpressed in human colon cancer. Dig Dis Sci 2005;50:42-6.**

**55. Roy HK, Iversen P, Hart J, Liu Y, Koetsier JL, Kim Y, et al. Down-regulation of SNAIL suppresses MIN mouse tumorigenesis: modulation of apoptosis, proliferation, and fractal dimension. Mol Cancer Ther 2004;3:1159-65.**

**56. Huang JC, Roy HK, Koetsier J, Kunte DP, Wali RK. Polyethylene glycol (PEG) mediated chemoprevention of colorectal cancer (CRC) involves downregulation of epidermal growth factor receptor (EGFR). Am J Gastroenterol 2005;100:S154-S154.**

**57. Torrance CJ, Jackson PE, Montgomery E, Kinzler KW, Vogelstein B, Wissner A, et al. Combinatorial chemoprevention of intestinal neoplasia. Nat Med 2000;6:1024-8.**

**58. Rosenberg DW. Genetic signatures of High- and Low-Risk Aberrant Crypt Foci in a Mouse Model of Sporadic Colon Cancer. Can Res 2004;64:6394-6401.**

**59. Bird RP. Observation and quantification of aberrant crypts in the murine colon treated with a colon carcinogen: preliminary findings. Cancer Lett 1987;37:147-51.**

**60. Glebov OK, Rodriguez LM, Soballe P, DeNobile J, Cliatt J, Nakahara K, et al. Gene expression patterns distinguish colonoscopically isolated human aberrant crypt foci from normal colonic mucosa. Cancer Epidemiol Biomarkers Prev 2006;15:2253-62.**

**61. Stevens RG, Swede H, Heinen CD, Jablonski M, Grupka M, Ross B, et al. Aberrant crypt foci in patients with a positive family history of sporadic colorectal cancer. Cancer Lett 2007;248:262-8.**

**62. Shpitz B, Bomstein Y, Mekori Y, Cohen R, Kaufman Z, Neufeld D, et al. Aberrant crypt foci in human colons: distribution and histomorphologic characteristics. Hum Pathol 1998;29:469-75.**

**63. Bouzourene H, Chaubert P, Seelentag W, Bosman FT, Saraga E. Aberrant crypt foci in patients with neoplastic and nonneoplastic colonic disease. Hum Pathol 1999;30:66-71.**

**64. Rudolph RE, Dominitz JA, Lampe JW, Levy L, Qu P, Li SS, et al. Risk factors for colorectal cancer in relation to number and size of aberrant crypt foci in humans. Cancer Epidemiol Biomarkers Prev 2005;14:605-8.**

**65. Seike K, Koda K, Oda K, Kosugi C, Shimizu K, Nishimura M, et al. Assessment of rectal aberrant crypt foci by standard chromoscopy and its predictive value for colonic advanced neoplasms. Am J Gastroenterol 2006;101:1362-9.**

**66. Wargovich MJ, Chen CD, Jimenez A, Steele VE, Velasco M, Stephens LC, et al. Aberrant crypts as a biomarker for colon cancer: evaluation of potential chemopreventive agents in the rat. Cancer Epidemiol Biomarkers Prev 1996;5:355-60.**

**67. Adler DG, Gostout CJ, Sorbi D, Burgart LJ, Wang L, Harmsen WS. Endoscopic identification and quantification of aberrant crypt foci in the human colon. Gastrointest Endosc 2002;56:657-62.**

**68. Moxon D, Raza M, Kenney R, Ewing R, Arozullah A, Mason JB, et al. Relationship of aging and tobacco use with the development of aberrant crypt foci in a predominantly African-American population. Clin Gastroenterol Hepatol 2005;3:271-8.**

**69. Gupta AK, Pretlow TP, Schoen RE. Aberrant crypt foci: what we know and what we need to know. Clin Gastroenterol Hepatol 2007;5:526-33.**

**70. Anti M, Marra G, Armelao F, Percesepe A, Ficarelli R, Ricciuto GM, et al. Rectal epithelial cell proliferation patterns as predictors of adenomatous colorectal polyp recurrence. Gut 1993;34:525-30.**

**71. Ahnen DJ, Byers T. Proliferation happens. Jama 1998;280:1095-6.**

**72. Paspatis GA, Zizi A, Chlouverakis GJ, Giannikaki ES, Vasilakaki T, Elemenoglou I, et al. Proliferative patterns of rectal mucosa as predictors of advanced colonic neoplasms in routinely processed rectal biopsies. Am J Gastroenterol 1998;93:1472-7.**

**73. Simanowski UA, Homann N, Knuhl M, Arce L, Waldherr R, Conradt C, et al. Increased rectal cell proliferation following alcohol abuse. Gut 2001;49:418-22.**

**74. Garewal H, Bernstein H, Bernstein C, Sampliner R, Payne C. Reduced bile acid-induced apoptosis in "normal" colorectal mucosa: a potential biological marker for cancer risk. Cancer Res 1996;56:1480-3.**

**75. Martin C, Connelly A, Keku TO, Mountcastle SB, Galanko J, Woosley JT, et al. Nonsteroidal anti-inflammatory drugs, apoptosis, and colorectal adenomas. Gastroenterology 2002;123:1770-7.**

**76. Zhang W, Gordon M, Lenz HJ. Novel approaches to treatment of advanced colorectal cancer with anti-EGFR monoclonal antibodies. Ann Med 2006;38:545-51.**

**77. Fichera A, Little N, Jagadeeswaran S, Dougherty U, Sehdev A, Mustafi R, et al. Epidermal growth factor receptor signaling is required for microadenoma formation in the mouse azoxymethane model of colonic carcinogenesis. Cancer Res 2007;67:827-35.**

**78. Cohen G, Mustafi R, Chumsangsri A, Little N, Nathanson J, Cerda S, et al. Epidermal growth factor receptor signaling is up-regulated in human colonic aberrant crypt foci. Cancer Res 2006;66:5656-64.**

**79. Malecka-Panas E, Kordek R, Biernat W, Tureaud J, Liberski PP, Majumdar AP. Differential activation of total and EGF receptor (EGF-R) tyrosine kinase (tyr-k) in the rectal mucosa in patients with adenomatous polyps, ulcerative colitis and colon cancer. Hepatogastroenterology 1997;44:435-40.**

**80. Pangburn HA, Kraus H, Ahnen DJ, Rice PL. Sulindac metabolites inhibit epidermal growth factor receptor activation and expression. J Carcinog 2005;4:16.**
